# Rapid and efficient generation of a transplantable population of functional retinal ganglion cells from fibroblasts

**DOI:** 10.1111/cpr.13550

**Published:** 2023-09-23

**Authors:** Zihui Xu, Yanan Guo, Kangjian Xiang, Dongchang Xiao, Mengqing Xiang

**Affiliations:** ^1^ State Key Laboratory of Ophthalmology Zhongshan Ophthalmic Center, Sun Yat‐sen University, Guangdong Provincial Key Laboratory of Ophthalmology and Visual Science Guangzhou China; ^2^ Guangdong Provincial Key Laboratory of Brain Function and Disease, Zhongshan School of Medicine Sun Yat‐sen University Guangzhou China

## Abstract

Glaucoma and other optic neuropathies lead to progressive and irreversible vision loss by damaging retinal ganglion cells (RGCs) and their axons. Cell replacement therapy is a potential promising treatment. However, current methods to obtain RGCs have inherent limitations, including time‐consuming procedures, inefficient yields and complex protocols, which hinder their practical application. Here, we have developed a straightforward, rapid and efficient approach for directly inducing RGCs from mouse embryonic fibroblasts (MEFs) using a combination of triple transcription factors (TFs): ASCL1, BRN3B and PAX6 (ABP). We showed that on the 6th day following ABP induction, neurons with molecular characteristics of RGCs were observed, and more than 60% of induced neurons became iRGCs (induced retinal ganglion cells) in the end. Transplanted iRGCs had the ability to survive and appropriately integrate into the RGC layer of mouse retinal explants and N‐methyl‐d‐aspartic acid (NMDA)‐damaged retinas. Moreover, they exhibited electrophysiological properties typical of RGCs, and were able to regrow dendrites and axons and form synaptic connections with host retinal cells. Together, we have established a rapid and efficient approach to acquire functional RGCs for potential cell replacement therapy to treat glaucoma and other optic neuropathies.

## Graphical abstract

The transcription factor (TF) combination ASCL1 + BRN3B + PAX6 could efficiently reprogram mouse embryonic fibroblasts (MEFs) into induced retinal ganglion cells (iRGCs). These iRGCs not only expressed several molecular markers of native RGCs, but also exhibited typical RGC electrophysiological properties. Upon transplantation into mouse retinal explants or NMDA‐damaged retinas, iRGCs could integrate into the ganglion cell layer, survive for a prolonged period, grow long neuron fibres, form synapses with host cells, and exhibit electrophysiological functions.

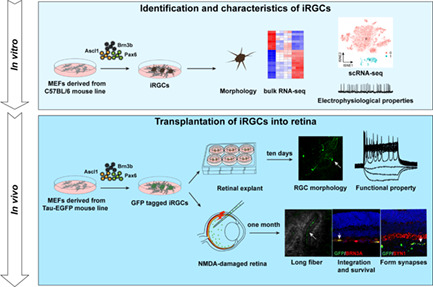

## 1 INTRODUCTION

The adult retina is composed of six types of neurons, rod, cone, bipolar, amacrine, horizontal and retinal ganglion cells (RGCs), and one type of glial cell (Müller glia), which work in coordination to ensure the precise transmission of visual signals.^1^ RGCs serve as the projection neurons of the retina and are accountable for the transmission of visual information from the eye to the brain. Glaucoma and other optic neuropathies are distinguished by the progressive loss of RGCs, leading to permanent vision impairment.^2^ While current therapies, including pharmacological and surgical treatments, can help to slow glaucoma disease progression by reducing intraocular pressure (IOP), they remain incapable of reversing blindness caused by RGC loss.^3^, ^4^

Cell replacement therapy is a promising approach for treating advanced glaucoma and other optic neuropathies. Previous studies have demonstrated that primary RGCs possess high vitality and, upon transplantation into rat retinas, can integrate into the ganglion cell layer (GCL), and form dendrites and synapses with host RGCs, allowing them to respond to light stimuli.^5^, ^6^ Nevertheless, the number of primary RGCs that can be isolated is incredibly limited, and the ethical concerns regarding obtaining primary human RGCs prohibit their clinical use.^2^ In recent years, the rapid development of stem cell technologies has provided an in vitro culture strategy for generating RGCs from embryonic stem cells (ESCs) or induced pluripotent stem cells (iPSCs).^7^, ^8^, ^9^ By mimicking in vivo retinal development, researchers add agonists and inhibitors of important signalling pathways involved in the regulation of retinal development at different periods of in vitro culture, such as Dickkopf‐1 (Dkk‐1; a Wnt inhibitor), Noggin (a bone morphogenetic protein inhibitor), DAPT (a Notch inhibitor), and so on, which can promote the directed differentiation of stem cells (ESCs/iPSCs) to RGCs.^10^, ^11^, ^12^ However, the method of obtaining RGCs from stem cells is still limited by the time‐consuming procedures (at least 3 weeks) and the risk of tumorigenesis due to incomplete differentiation after transplantation.^2^, ^10^, ^13^, ^14^

In comparison, transcription factor (TF)‐mediated direct reprogramming strategy offers a more direct and rapid route for generating desired cell types. This approach has been successfully applied in the reprogramming of fibroblasts into various cell types such as neurons, cardiomyocytes, neural stem cells, photoreceptors and corneal endothelial cells.^15^, ^16^, ^17^, ^18^, ^19^, ^20^ Recently, we successfully reprogrammed fibroblasts directly into sensory ganglion (SG) neurons and induced retinal ganglion cells (iRGCs) in vitro using a combination of TFs ASCL1, BRN3B/3A and ISL1 (ABI).^21^ In addition, another group followed up by demonstrating that ABI could induce Müller cells into a small number of RGC‐like cells in the adult mouse retina.^22^ However, iRGCs induced by ABI only accounted for ~12% of the induced neurons (iNs), indicating a relatively low efficiency of iRGC induction by this TF combination.^21^

In this study, we screened for and identified a novel combination of TFs, ASCL1, BRN3B and PAX6 (ABP), that could rapidly and efficiently reprogram mouse embryonic fibroblasts (MEFs) into iRGCs. Our results showed that more than 60% of iNs co‐expressed TUJ1 and the RGC marker BRN3A, as well as other RGC‐specific markers such as THY1.2, RBPMS, SNCG or ISL1. Moreover, upon transplantation, ABP‐induced RGCs not only properly integrated into the GCL of mouse retinal explants and N‐methyl‐d‐aspartic acid (NMDA)‐damaged retinas, but also formed dendrites, axons and synapses with the host retina.

## 2 RESULTS

### 2.1 Screening for TFs that efficiently reprogram fibroblasts into iRGCs

The fate of various retinal cell types is determined by both intrinsic and extrinsic factors, with TFs playing a predominant role as intrinsic factors in regulating this process.^23^ The regulation of retinal development has been explored for decades, and currently the TFs responsible for determining the fate of each type of retinal cells have been identified. Combining findings from our group with others on the transcriptional regulation of RGCs,^24^ we selected a total of six TFs, including five RGC TFs (BRN3B, DLX1, EBF1, ISL1 and PAX6) and a neural TF (ASCL1), for an in vitro study of RGC induction. TUJ1 and BRN3A are common specific markers for neurons and RGCs, respectively, and were used as markers for preliminary screening of iRGCs.^25^

**FIGURE 1:**
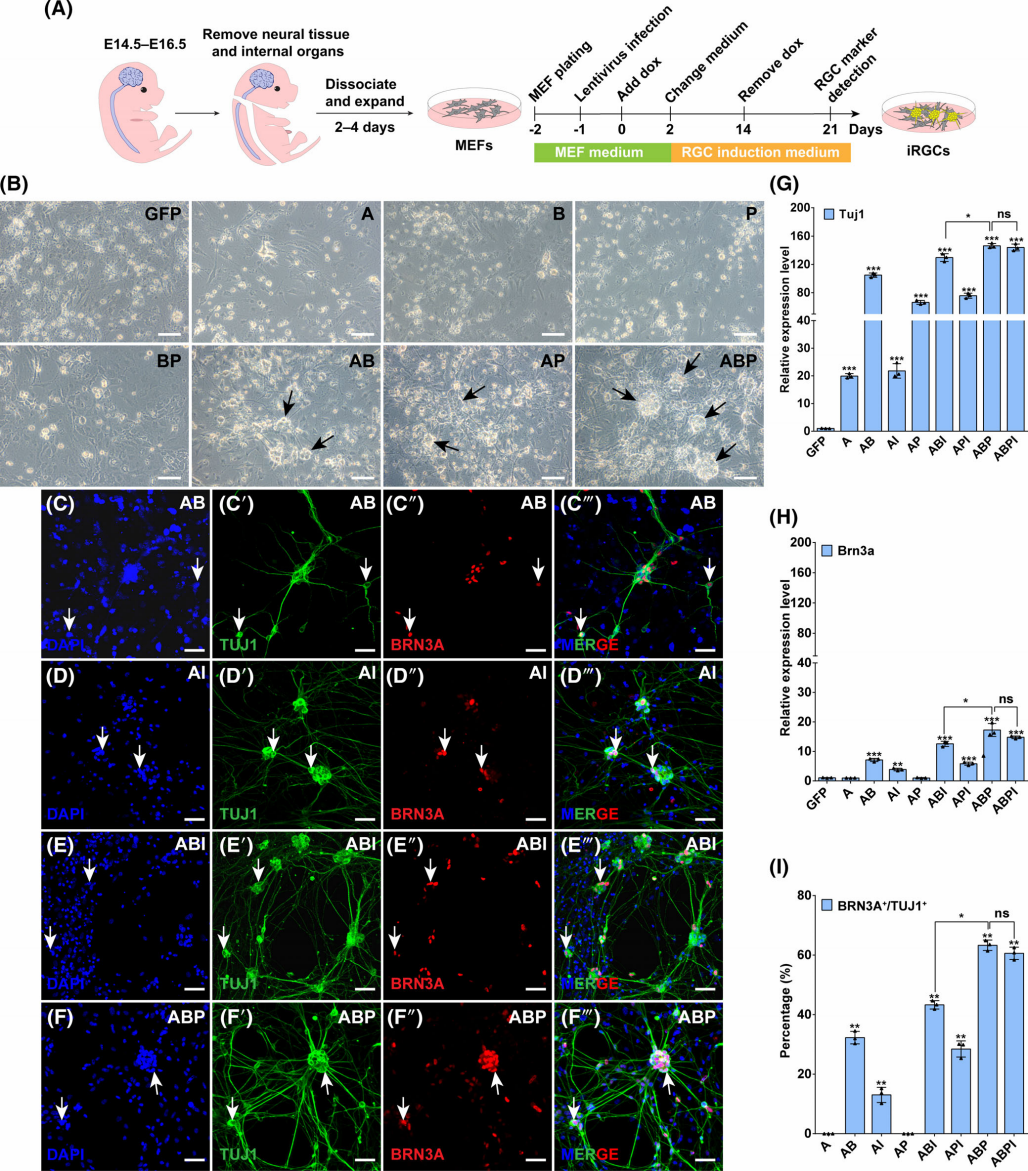
Efficient conversion of MEFs into iRGCs by ABP. (A) Schematic illustration of the process of TF‐induced conversion of RGCs from MEFs. (B) Bright‐field images showing morphological changes of MEFs infected with the indicated lentiviruses (A, ASCL1; B, BRN3B; P, PAX6) and cultured for 21 days (D21). Arrows point to neuronal clusters. Scale bars, 80 μm. (C–C‴, D–D‴, E–E‴, F–F‴) Double‐immunostaining for TUJ1 and BRN3A in MEFs on D21 after infection with the indicated lentiviruses. Nuclei were stained with DAPI. Arrows indicate colocalized cells. Scale bars, 40 μm. (G, H) qRT‐PCR analyses showing relative expression levels of the *Tuj1* and *Brn3a* genes in MEFs infected with the indicated lentiviruses on D21 post induction. Data are presented as mean ± SD (*n* = 3). Asterisks indicate significance in one‐way ANOVA test: *p < 0.05, **p < 0.001, ***p < 0.0001. (I) Quantification of TUJ1‐positive cells that express BRN3A in MEFs infected with the indicated lentiviruses on D21. Data are presented as mean ± SD (*n* = 3). Asterisks indicate significance in one‐way ANOVA test: *p < 0.001, **p < 0.0001. ns, no significance.

In order to obtain a combination of TFs efficient for inducing RGCs, we prepared six lentiviruses containing single TFs and infected MEFs using different combinations (Figures 1A and S1A–C). We established a control group by infecting MEFs with the GFP lentivirus, and confirmed that they did not express any neuronal or RGC markers (Figure S2). Among the six single TF groups, only ASCL1 (A) was able to induce TUJ1‐positive cells, but no BRN3A‐positive cell was observed in any of the six groups (Figure S1A,F–K), and there was no obvious change in cell morphology compared with the control group (Figure 1B). To induce BRN3A‐positive cells, we co‐infected MEFs with lentiviruses expressing ASCL1 (A) and each of the following factors: BRN3B (B), DLX1 (D), EBF1 (E), ISL1 (I) and PAX6 (P). On day (D) 21 after lentivirus infection, neuronal clusters were observed in MEFs infected with AB, AI and AP (Figures 1B,C–C‴,D–D‴ and S1O), resembling ganglion‐like structures also observed in the in vitro 2D differentiation of RGCs from ESCs.^14^, ^26^ Moreover, while double‐TF‐reprogrammed cells in general showed a significant increase in *Tuj1* mRNA levels compared with single‐TF‐reprogrammed ones (Figures 1G and S1D), only MEFs infected with AB or AI displayed co‐expression of TUJ1 and BRN3A, marking RGC‐like cells (Figures 1C–C‴,D–D‴ and S1B,L–O). In these two groups, among iNs labelled with TUJ1, 32.28% ± 1.22% and 13.04% ± 2.55%, respectively, expressed BRN3A (Figure 1I), suggesting that BRN3B and ISL1 may be indispensable factors in the in vitro induction of iRGCs, whose roles in determining the fate of mouse RGCs have been confirmed in earlier studies.^27^, ^28^

To determine whether further improvement in RGC induction efficiency was possible, we infected MEFs with combinations of lentiviruses expressing three TFs (Figure S1C). Quantitative real‐time PCR (qRT‐PCR) analysis revealed that three‐factor combinations in general further increased the transcription levels of *Tuj1* and *Brn3a*, with the ABP‐reprogrammed cells exhibiting the highest expression (Figures 1G,H and S1D,E). Immunofluorescence staining and quantification of iNs induced by ABI, API and ABP revealed that among TUJ1‐positive iNs, the proportions of iRGCs (BRN3A^+^TUJ1^+^) were 43.3% ± 1.4%, 28.45% ± 2.7%, and 63.3% ± 1.75%, respectively, and ABPI did not further improve the induction efficiency (Figures 1E–E‴,F–F‴,I and S1P–U). These data indicate that ABP can efficiently induce MEFs to generate iRGCs.

### 2.2 iRGCs exhibit molecular characteristics of retinal ganglion cells

**FIGURE 2:**
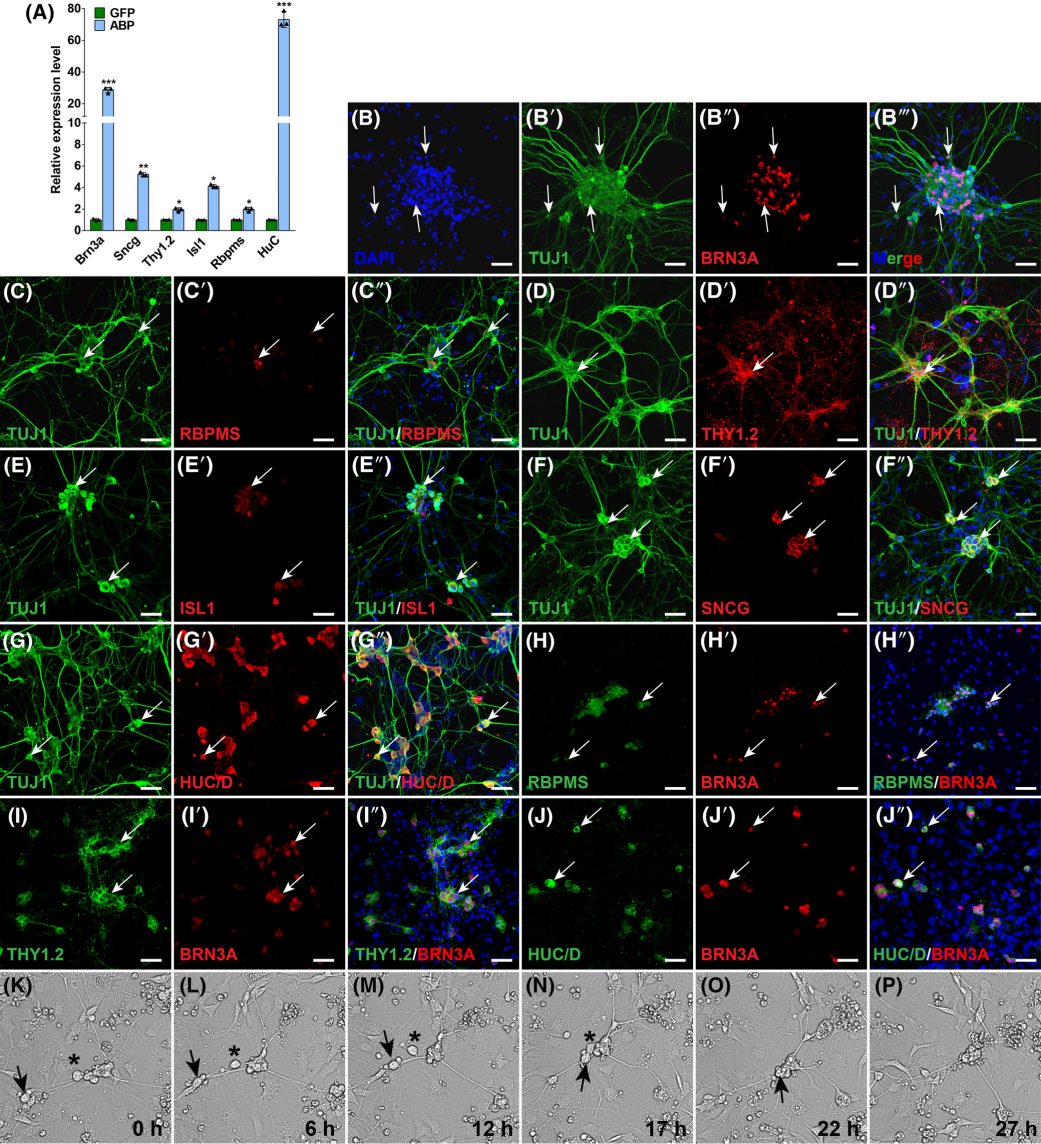
Characterization of ABP‐reprogrammed iRGCs. (A) qRT‐PCR analysis showing relative expression levels of RGC‐related makers in GFP‐ and ABP‐transduced MEFs. Data are presented as mean ± SD (*n* = 3). Asterisks indicate significance in unpaired two‐tailed Student's *t* test: *p < 0.05, **p < 0.001, ***p < 0.0001. (B–B‴, C–C″, D–D″, E–E″, F–F″, G–G″) ABP‐reprogrammed iRGCs were double‐immunostained for TUJ1 and RGC makers BRN3A, RBPMS, THY1.2, ISL1, SNCG or HUC/D. Nuclei were stained with DAPI. Scale bars, 40 μm. Arrows indicate colocalized cells. (H–H″, I–I″, J–J″) ABP‐reprogrammed iRGCs were double‐immunostained for BRN3A and RGC makers RBPMS, THY1.2, or HUC/D. Nuclei were stained with DAPI. Arrows indicate colocalized cells. Scale bars, 40 μm. (K–P) Snapshots of a time‐lapse video showing how iRGCs induced by ABP aggregated. The arrow and the asterisk indicate the positions of two individual iRGCs at different time points.

Immunofluorescence staining of adult and E14.5 mouse retinas showed that BRN3A, RBPMS and THY1.2 were specifically expressed in RGCs (Figure S3B–B″,D–D″,E–G). Although SNCG and ISL1 were expressed in other retinal cells of the inner nuclear layer, they were expressed in RGCs and showed considerable co‐localization with the RGC‐specific marker BRN3A (Figure S3A–A″,C–C″,H,I). In addition, previous studies have shown that HUC/D (ELAVL3/4) is highly expressed in subtypes of mouse RGCs.^29^ Therefore, BRN3A, RBPMS, THY1.2, SNCG, ISL1 and HUC/D can serve as molecular markers for identifying iRGCs. First, we used qRT‐PCR to measure the mRNA levels of *Brn3a*, *Sncg*, *Thy1.2*, *Isl1*, *Rbpms* and *HuC* in MEFs infected with ABP, ABD, AIE or GFP lentiviruses, and the results showed that the expression of these molecular markers was significantly higher in ABP‐transduced MEFs than that in ABD‐, AIE‐ or GFP‐transduced MEFs; in addition, *Thy1.2*, *Isl1* and *Rbpms* were not expressed in ABD or AIE‐transduced MEFs (Figures 2A and S3R,S). We then carried out double‐immunofluorescence staining of ABP‐transduced MEFs with antibodies against these proteins and the neuronal marker TUJ1, and observed numerous co‐localizations (Figure 2B–B‴,C–C″,D–D″,E–E″,F–F″,G–G″), while ABI‐transduced MEFs only expressed THY1.2 and SNCG, and API‐transduced MEFs only expressed SNCG (Figure S3J–J″,K–K″,L–L″,M–M″,N–N″,O–O″,P–P″,Q–Q″). Furthermore, co‐staining for RBPMS, THY1.2 or HUC/D with BRN3A in iRGCs also identified numerous positive cells co‐labelled for double markers (Figure 2H–H″,I–I″,J–J″), indicating that ABP‐induced RGCs are molecularly characteristic of native RGCs.

Bright‐field images showed that ABP‐reprogrammed neurons had a tendency toward aggregating into clusters (Figure 1B). Moreover, most RGC markers were expressed in cells that formed neuron clusters. This phenomenon was also observed in RGCs differentiated from retinal precursor cells and human iPSCs in vitro,^14^, ^26^ which suggests that the formation of neuron clusters may be a preferential behaviour of RGCs cultured in vitro. To investigate how ABP‐reprogrammed neurons aggregated into iRGC clusters, we used long‐term time‐lapse microscopy to track their behaviour over time in culture. Specifically, we induced MEFs with ABP or ASCL1 lentiviruses for a period of 10 days before recording. Our observations revealed that compared with ASCL1‐iNs, the ABP‐iNs displayed a more rounded shape with the presence of more neurites (Videos S1 and S2). Over a period of 50 h, the ABP‐iNs migrated, subsequently formed small clusters, and continued to coalesce and grow into bigger ones closely resembling RGC clusters (Figure 2K–P; Video S1). We did not observe a similar self‐organization phenomenon for iNs induced by ASCL1 (Video S2).

**FIGURE 3:**
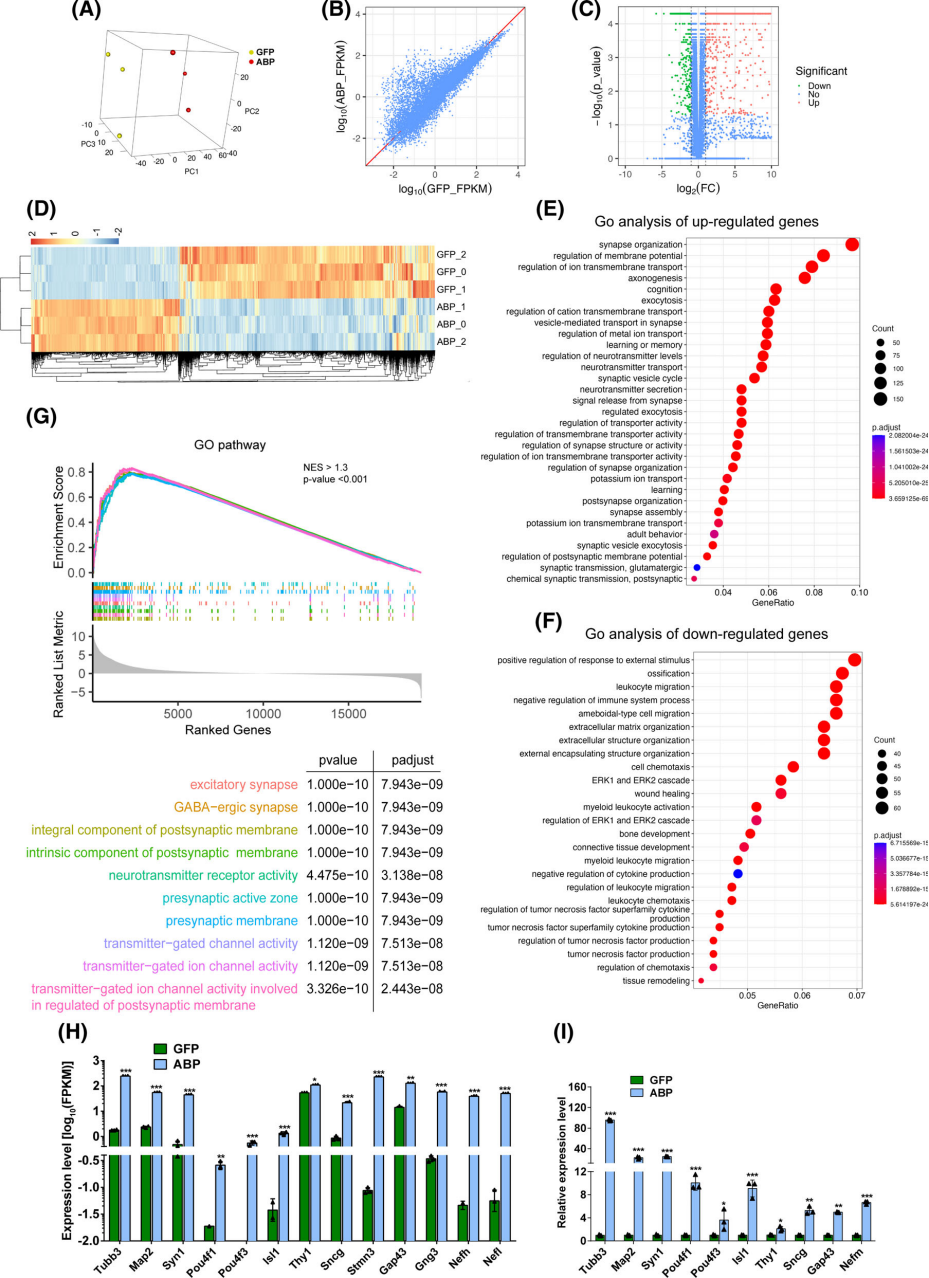
Global gene expression profiles of GFP‐ and ABP‐transduced MEFs (D21) determined by bulk RNA‐seq analysis. (A) 3D principal component analysis (PCA) of the global gene expression profiles of GFP‐ and ABP‐transduced MEFs. (B) Scatter plot analysis of the global gene expression profiles of GFP‐ and ABP‐transduced MEFs. (C) Volcano plot (significance vs. fold change) of significantly altered genes (fold change ≥2 and *p*‐value <0.05) between GFP‐ and ABP‐transduced MEFs. (D) Heatmap of the *z*‐transformed expression values of differentially expressed genes between ABP‐ and GFP‐transduced MEFs. (E,F) GO enrichment analysis of upregulated or downregulated genes in ABP‐transduced MEFs compared with GFP‐transduced ones. (G) Top 10 GO terms, revealed by gene set enrichment analysis (GSEA), which the upregulated genes are enriched for. (H) Expression levels (FPKM) of the indicated significantly upregulated genes in ABP‐transduced MEFs compared with GFP‐transduced ones, which represent RGC‐related markers. Data are presented as mean ± SD (*n* = 3). Asterisks indicate significance in unpaired two‐tailed Student's *t* test: *p < 0.05, **p < 0.001, ***p < 0.0001. (I) qRT‐PCR analysis of the relative expression levels of RGC‐related marker genes in GFP‐ and ABP‐transduced MEFs. Data are presented as mean ± SD (*n* = 3). Asterisks indicate significance in unpaired two‐tailed Student's *t* test: *p < 0.05, **p < 0.001, ***p < 0.0001.

To further confirm the iRGC identity, we conducted by bulk RNA‐seq a transcriptome profiling analysis of ABP‐ and GFP‐transduced MEFs on D21. Principal component analysis (PCA) revealed that the three replicates of ABP‐ and GFP‐transduced MEFs each formed a separate cluster, indicating good reproducibility of the sequencing samples and significant differences in their transcriptomes (Figure 3A). Scatter plot of gene expression levels between ABP‐ and GFP‐transduced MEFs confirmed that there were substantial differences between the two groups (Figure 3B). Analysis of differentially expressed genes (fold change ≥2 and *p*‐value <0.05) revealed that ABP‐transduced MEFs had 887 downregulated genes and 2926 upregulated genes compared with GFP‐transduced ones. Among the upregulated genes were a number of molecular markers for RGCs, including *Pou4f1* (*Brn3a*), *Pou4f2* (*Brn3b*), *Isl1*, *Sncg*, *Elavl3* (*HuC*) and so on (Figure 3C; Table S3.). Consistent with these results, hierarchical cluster analyses also showed that numerous genes were either up‐ or down‐regulated in ABP‐transduced MEFs compared with GFP‐transduced ones (Figure 3D). GO enrichment analysis showed that these upregulated genes were enriched for those associated with neuronal functions such as synapse organization, vesicle‐mediated transport in synapse, neurotransmitter transport, synaptic vesicle cycle, regulation of neurotransmitter levels, neurotransmitter secretion and signal release from synapse (Figure 3E), and that the downregulated genes were associated with fibroblast properties such as extracellular matrix/structure organization, cell migration and cell chemotaxis (Figure 3F). Consistent with these results, gene set enrichment analysis (GSEA) also showed that the upregulated genes were enriched for those associated with synapse organization such as excitatory synapse, postsynaptic membrane, presynaptic membrane and so on (Figure 3G). qRT‐PCR assays were carried out to validate the bulk RNA‐seq results and further confirmed the up‐regulation of numerous neuronal and RGC‐specific genes in ABP‐transduced MEFs, including *Tubb3* (*Tuj1*), *Map2*, *Syn1* (Synapsin 1), *Pou4f1*, *Pou4f3* (*Brn3c*), *Isl1*, *Sncg*, *Thy1* and *Gap43* (Figure 3H,I).

### 2.3 iRGCs are glutamatergic neurons

To determine whether ABP‐reprogrammed iRGCs are excitatory or inhibitory, we performed immunofluorescence staining of ABP‐transduced MEFs on D21. The results showed that ABP‐induced RGCs expressed both MAP2 and Synapsin 1 proteins, indicating that iRGCs at this stage had mature neuronal properties (Figure S4A–C). Previous studies confirmed the expression of glutamatergic neuron markers VGLUT1, VGLUT2 and VGLUT3 in RGCs,^30^, ^31^ which is consistent with our immunofluorescence staining results from the adult mouse retina (Figure S4D–F). Furthermore, there were VGLUT1‐, VGLUT2‐ and VGLUT3‐expressing cells in ABP‐induced neuron clusters, but no cells immunoreactive for dopaminergic neuron marker TH or GABAergic neuron marker GABA (Figure S4G–L). These immunolabelling results were consistent with those of qRT‐PCR assays which showed that ABP upregulated the expression of *Vglut1–3* but caused no expression change of *Th*, *Gad1* or the glycinergic transporter gene *Glyt1* (Figure S4M). These data together suggest that ABP‐induced RGCs are glutamatergic neurons as most native RGCs.

### 2.4 iRGCs exhibit gradually mature electrophysiological characteristics

**FIGURE 4:**
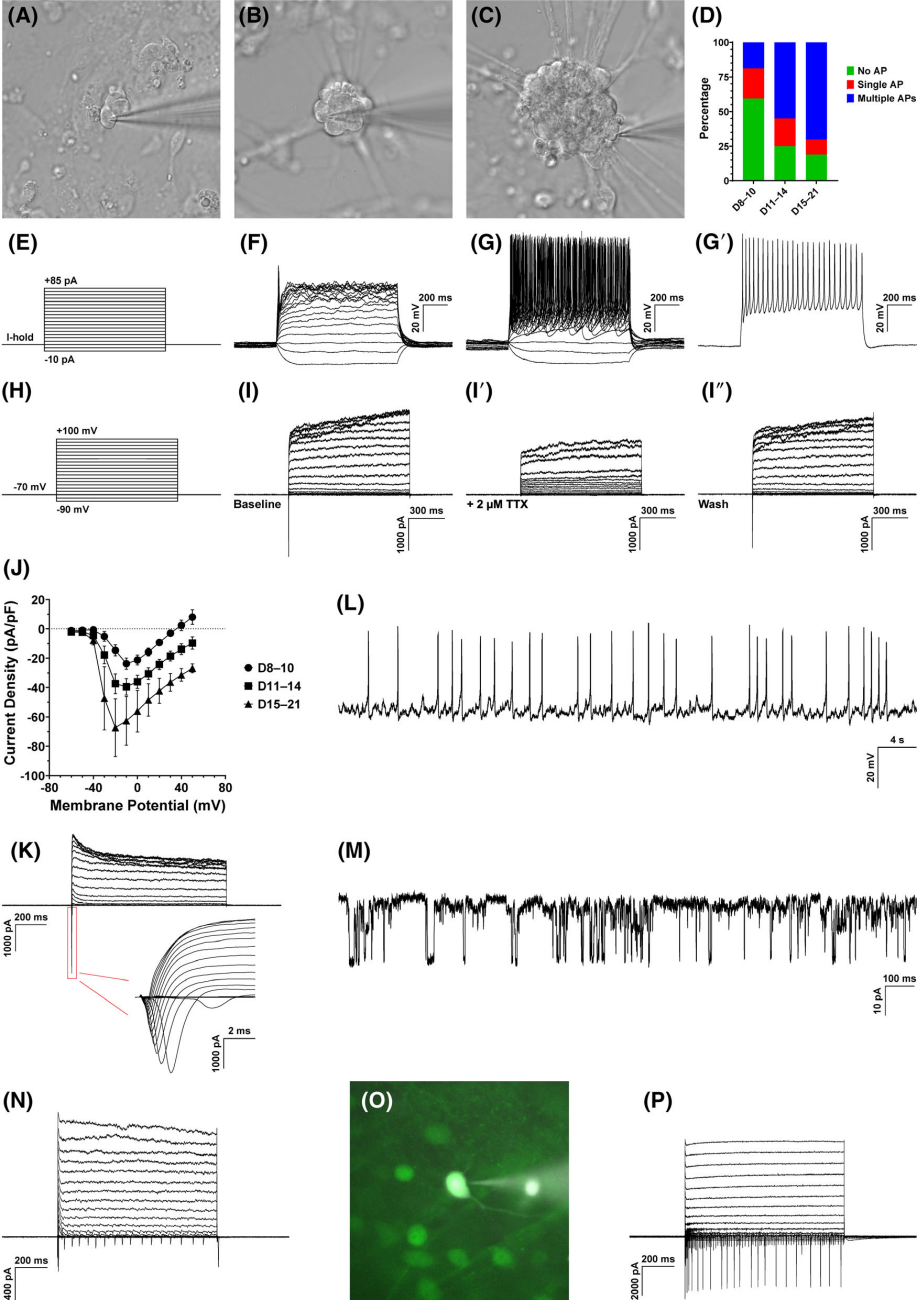
iRGCs display the electrophysiological properties typical of native RGCs. (A–C) Bright‐field images showing the neurons chosen for patch‐clamp recordings at different stages (D8, 14 and 21). (D) Observed ratios of iRGCs that were multiple‐spiking, single‐spiking, or displayed no action potential (AP). D8–10, *n* = 32; D11–14, *n* = 40; D15–21, *n* = 37. (E) The waveform of the stepwise current injection protocol. I‐hold: a holding current injected to maintain the initial membrane potential at ~65 mV. (F) Current injection revealed a single AP response (single‐spiking) of an iRGC. (G,G′) Current injection revealed multiple AP responses (multiple‐spiking) from another iRGC. (H) The waveform of the stepwise voltage injection protocol. (I–I″) Whole‐cell current recording showed fast activated and inactivated inward sodium currents of an iRGC. The sodium currents could be completely inhibited by 2 μM tetrodotoxin (TTX) (I′), and recover after TTX removal (I″). (J) The current–voltage curve of iRGCs recorded at different stages. Data are presented as mean ± SD. D8–10, *n* = 11; D11–14, *n* = 27; D15–21, *n* = 24. (K) An iRGC exhibiting fast inward sodium currents and outward potassium currents. (L) Spontaneous APs (sAPs) recorded from an iRGC. (M) Spontaneous post synaptic currents (sPSCs) recorded from an iRGC. (N) Whole‐cell current recording showed multiple inward sodium current spikes of an iRGC. (O) Epifluorescent image of an RGC from the *Brn3b*‐GFP mouse retina chosen for recording. (P) Whole‐cell current recording showed multiple inward sodium current spikes of the *Brn3b*‐GFP mouse RGC.

To assess the electrophysiological characteristics of iRGCs reprogrammed from MEFs by ABP, we performed whole‐cell patch‐clamp recordings of cells with neuronal morphology. Consistent with previous findings, we observed the formation of neuron clusters that grew in size over time (Figure 4A–C). By as early as D8, the majority of recorded iRGCs generated potassium currents and small sodium currents (Figure 4J) but no action potential (AP), suggesting that they were functionally immature. While approximately 60% of iRGCs recorded during D8–10 was incapable of generating APs, about 21% of iRGCs fired a typical single AP spike (Figure 4D–F) and about 19% were already multi‐spiking (Figure 4D). As iRGCs became mature, the proportion of multi‐spiking cells increased, constituting around 55% of cells recorded during D11–14 and around 70% during D15–21 (Figure 4D,G,G′), accompanied by increasing amplitudes of fast‐activating inward sodium currents (Figure 4H,J,K). The inward sodium currents were selectively inhibited by tetrodotoxin (TTX) and completely restored upon its removal (Figure 4I–I″). Some recorded iRGCs were capable of firing spontaneous APs (sAPs) (Figure 4L), and some exhibited spontaneous postsynaptic currents (sPSCs) (Figure 4M) in agreement with the synapsin immunoreactivity (Figure S4A–C), implying the formation of functional synapses between iRGCs.

We previously generated a mouse line by inserting the GFP sequence into the *Brn3b* (*Pou4f2*) gene locus, so that all BRN3B‐expressing RGCs are GFP‐tagged. In order to compare the electrophysiological properties between iRGCs and native RGCs, we also performed patch‐clamp recordings on GFP‐expressing RGCs from *Brn3b*‐GFP mouse retinas (Figure 4O), and found that most of these native RGCs (6 out of 9 cells in 6 animals) generated multiple spikes of inward sodium currents in response to depolarizing voltage steps (Figure 4P). Similarly, more than half of iRGCs (13 out of 24) recorded during D15–21 generated multiple spikes of inward sodium currents (Figure 4N). Therefore, iRGCs induced by ABP gradually mature and possess the membrane and electrophysiological properties typical of native RGCs.

### 2.5 ABP induces rapid direct reprogramming of MEFs into iRGCs

**FIGURE 5:**
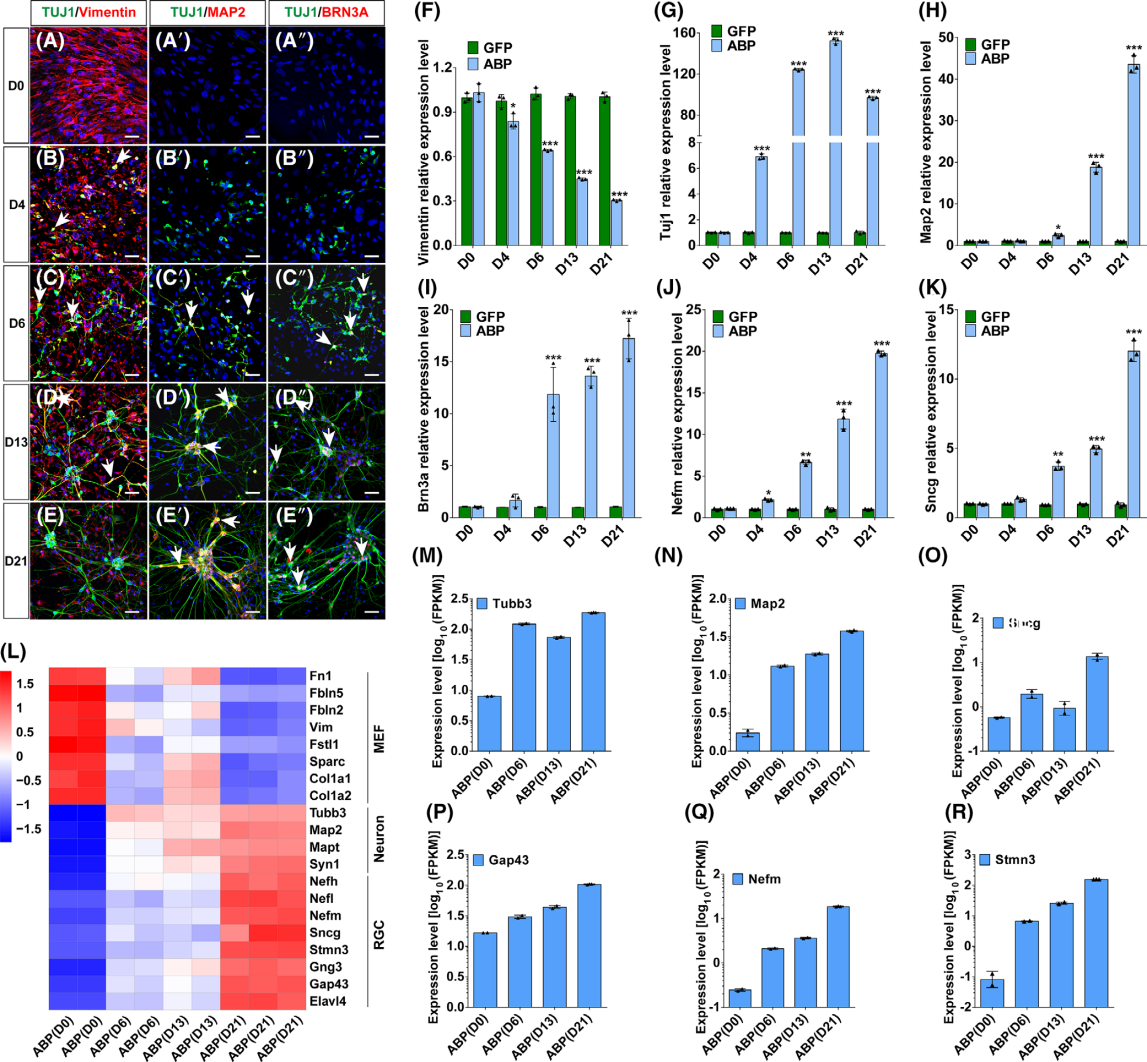
Quick conversion of MEFs into iRGCs by ABP. (A–E, A′–E′, A″–E″) Immunofluorescence staining of ABP‐transduced MEFs with the indicated antibodies at the indicated time points. Nuclei were stained with DAPI. Arrows indicate colocalized cells. Scale bars, 40 μm. (F–K) qRT‐PCR analysis showing the time course (D0–21) of relative expression levels of the fibroblast marker gene Vimentin, neuronal marker genes *Tuj1* and *Map2*, and RGC marker genes *Brn3a*, *Nefm* and *Sncg* in GFP‐ and ABP‐transduced MEFs. Data are presented as mean ± SD (*n* = 3). Asterisks indicate significance in unpaired two‐tailed Student's *t* test: *p < 0.05, **p < 0.001, ***p < 0.0001. (L) Expression heatmap of representative differentially expressed genes among samples (2 or 3 at each time point) at the indicated time points. (M–R) Expression levels (FPKM) of the indicated significantly upregulated genes in ABP‐transduced MEFs compared with GFP‐transduced ones at the indicated time points.

To determine the onset of ABP‐induced MEF transdifferentiation into iRGCs, we performed immunofluorescence staining and qRT‐PCR to detect fibroblast marker Vimentin, neuronal markers *Tuj1* and *Map2*, and RGC markers *Brn3a*, *Nefm* and *Sncg* at mRNA and/or protein levels on D0, 4, 6, 13 and 21. We observed a decrease in Vimentin expression and a significant increase in early neuronal marker *Tuj1* expression starting at D4 (Figure 5A–A″,B–B″,F,G). Although mature neuronal marker *Map2* and RGC markers *Brn3a*, *Nefm* and *Sncg* did not show a significant increase at this time point, there was an upward trend in their expression levels (Figure 5H–K). On D6, they all exhibited high levels of expression, and continued to increase from that point forward (Figure 5H–K). Additionally, TUJ1 and Vimentin co‐localized almost entirely on D4 (Figure 5B). Over time, the number of co‐localized cells gradually decreased (Figure 5B–D) and by D21, there was no longer co‐localization (Figure 5E). Meanwhile, TUJ1^+^MAP2^+^ and TUJ1^+^BRN3A^+^ double‐positive cells appeared on D6 and gradually increased from D6 to D21 (Figure 5C′–E′,C″–E″). These observations indicate a rapid transition of MEFs through an intermediate state expressing both fibroblast and neuronal markers toward neurons following ABP lentivirus infection.

To further characterize the rapid induction process of ABP‐induced iRGCs, bulk RNA‐seq was performed on MEFs infected with ABP lentiviruses on D0 [ABP(D0)], D6 [ABP(D6)], D13 [ABP(D13)] and D21 [ABP(D21)]. PCA analysis showed the biggest difference between ABP(D0) and ABP(D21) samples, while ABP(D6) and ABP(D13) samples showed smaller differences (Figure S5A), which was consistent with the results of hierarchical clustering analysis (Figure S5B). Furthermore, heatmap analysis showed that the expression of fibroblast marker genes *Fn1*, *Fbln5*, *Fbln2*, *Vim* (Vimentin) and *Fstl1* gradually decreased over time, while the expression of neuronal marker genes *Tubb3*, *Map2*, *Mapt* (*Tau*) and *Syn1*, and RGC marker genes *Sncg*, *Gap43*, *Nefm* and *Stmn3* increased (Figure 5L–R). In agreement with these results, volcano plots and Venn diagram analysis showed that the expression of RGC marker genes *Pou4f1*, *Gng3*, *Stmn3* and *Nefl*, among others, began on D6, and the expression levels increased over time (Figure S5C–F).

To ascertain whether the induction of iRGCs by ABP from MEFs underwent direct conversion or proliferative intermediate stages, we pulse‐labelled cells with 5‐ethynyl‐2′‐deoxyuridine (EdU) for 24 h on D20 and found that almost no TUJ1‐positive cells were labelled by EdU, whereas approximately 5% of TUJ1‐negative cells (e.g., MEFs) were labelled (Figure S6A,D–D″,E). We then reprogrammed MEFs with ABP in the presence of EdU for 19 days starting from D2. In this case, only 0.57% of TUJ1‐positive cells were labelled by EdU, while 64.6% of TUJ1‐negative cells were labelled (Figure S6A,B–B″,C). These results suggest that iRGCs are most likely induced by direct cell reprogramming without undergoing a proliferative intermediate state.

### 2.6 Characterization of iRGCs by scRNA‐seq profiling

**FIGURE 6:**
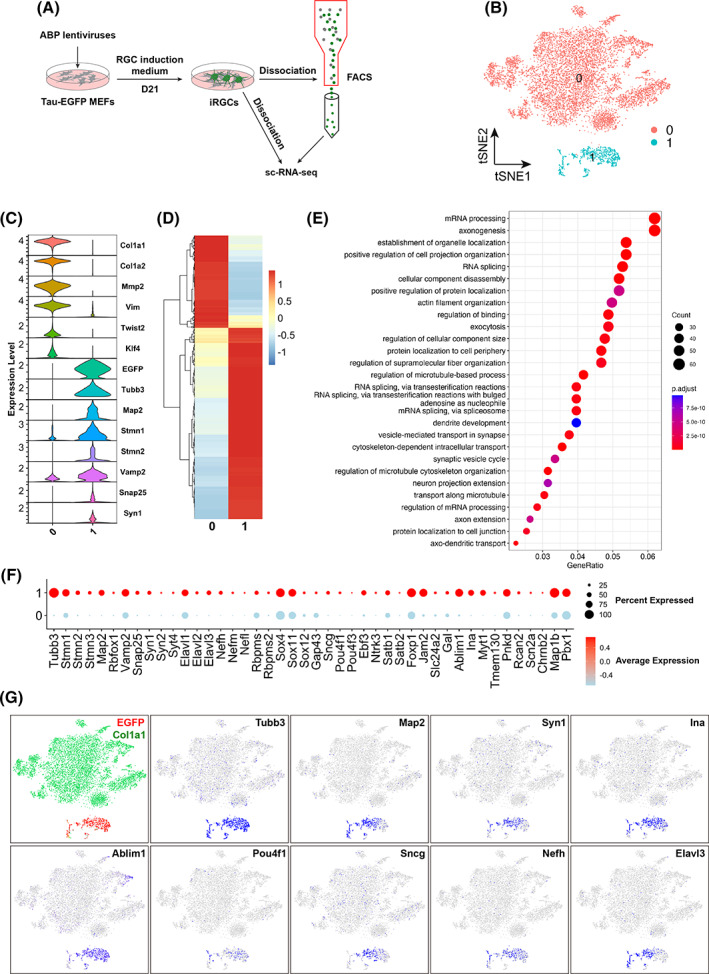
Characterization of ABP‐induced iRGCs by scRNA‐seq profiling. (A) Schematic illustration of the process for scRNA‐seq analysis. The cells were prepared with or without fluorescence‐activated cell sorting (FACS) before sequencing. (B) Two cell clusters were identified by t‐distributed Stochastic Neighbour Embedding (t‐SNE) plot analysis. (C) Stacked violin plots showing the expression patterns of the MEF marker genes (*Col1a1*, *Col1a2*, *Mmp2*, *Vim*, *Twist2* and *Klf4*), neuronal marker genes (*Tubb3*, *Map2*, *Stmn1*, *Stmn2*, *Vamp2*, *Snap25* and *Syn1*), and reporter *EGFP* gene in the two clusters. (D) Expression heatmap of the differentially expressed genes between clusters 1 and 0. (E) Gene ontology (GO) enrichment analysis of the upregulated genes between clusters 1 and 0. (F) Dot plots showing high expression of neuron and RGC‐specific genes in cell cluster 1. (G) t‐SNE plots coloured by expression of the indicated reporter, MEF marker, neuronal marker, and RGC marker genes.

To further characterize ABP‐induced iRGCs, single‐cell transcriptome profiling was performed. Single ABP‐transduced Tau‐EGFP MEFs or FACS (fluorescence‐activated cell sorting)‐sorted ABP‐induced GFP‐tagged iRGCs were sequenced using the 10× Genomics Chromium platform (Figure 6A).^32^ After processing the sequencing data of ABP‐transduced Tau‐EGFP MEFs by the Cell Ranger software pipeline, we grouped the 11,386 sequenced single cells into two clusters using the Seurat software package (Figure 6B), an R toolkit for single‐cell genomics.^33^ Stacked violin plot shows high levels of expression of *EGFP*, and neuronal marker genes *Tubb3*, *Map2*, *Stmn1*, *Stmn2*, *Vamp2*, *Snap25* and *Syn1* in cluster 1, whereas the expression of fibroblast marker genes *Col1a1*, *Col1a2*, *Mmp2*, *Vim*, *Twist2* and *Klf4* are limited to cluster 0 (Figures 6C), suggesting that cluster 1 represents iNs and cluster 0 MEFs. Hierarchical cluster analyses revealed numerous differentially expressed genes, displaying either up‐regulation or down‐regulation between clusters 1 and 0 (Figure 6D). Gene ontology (GO) analysis of the upregulated genes indicated their enrichment in processes such as axonogenesis, dendrite development, synapse organization, vesicle‐mediated transport in synapse, synaptic vesicle cycle, axon extension and so on (Figures 6E). Consistent with these results, dot and feature plots further revealed the upregulation of a series of neuronal marker genes and in particular RGC marker genes such as *Ina*, *Ablim1*, *Pou4f1* (*Brn3a*), *Sncg*, *Nefh* and *Elavl3* (*Huc*) in cluster 1 (Figure 6F,G), indicating that cluster 1 represents a population of reprogrammed iRGCs.

To further explore the difference/similarity between the iRGCs induced by ABP and iSG (induced sensory ganglion) neurons induced by ABI (ASCL1 + BRN3B/3A + ISL1), we integrated the current iRGC scRNA‐seq dataset with the previous iSG scRNA‐seq dataset using Seurat. The integration analysis yielded 17 mostly distinct and non‐overlapping cell clusters between the iRGCs and iSG neurons (Figure S7A–C). While both cell types express general neuronal marker genes such as *Tubb3*, *Stmn1* and *Vamp2*, RGC‐specific genes such as *Sncg*, *Jam2* and *Myt1* are highly expressed only in ABP‐induced iRGCs (Figure S7D). Similarly, there are a set of SG‐specific genes such as *Gal*, *Ntrk3* and *P2rx3* that are highly expressed only in iSG neurons (Figure S7D). These results imply that ABP and ABI reprogram MEFs into different types of neurons.

### 2.7 iRGCs survive and integrate into retinal explants and damaged retinas after transplantation

We next determined whether ABP‐induced RGCs were able to survive and integrate into host retinas after transplantation. To track transplanted cells, we used MEFs derived from the Tau‐EGFP mouse line, whose RGCs and their axons are GFP‐positive (Figure S8A).^34^, ^35^ In agreement, our immunofluorescence staining of Tau‐EGFP retinas showed extensive co‐labelling of GFP with the neuronal marker TUJ1 or the RGC marker BRN3A in the GCL (Figure S8B–B″,C). Moreover, TAU and BRN3A were also co‐expressed in RGCs of the C57BL/6 mouse retinas (Figure S8D). In ABP‐transduced Tau‐EGFP MEFs, many GFP‐labelled clusters were observed on D21 (Figure S8E–E″). Immunofluorescence staining showed that these GFP‐positive cells co‐expressed not only neuronal markers TUJ1 and MAP2 (Figure S8F–F‴), but also RGC markers THY1.2, BRN3A and RBPMS (Figure S8G–G‴,H–H‴), suggesting that a substantial number of GFP‐positive iRGCs were generated from ABP‐transduced Tau‐EGFP MEFs.

**FIGURE 7:**
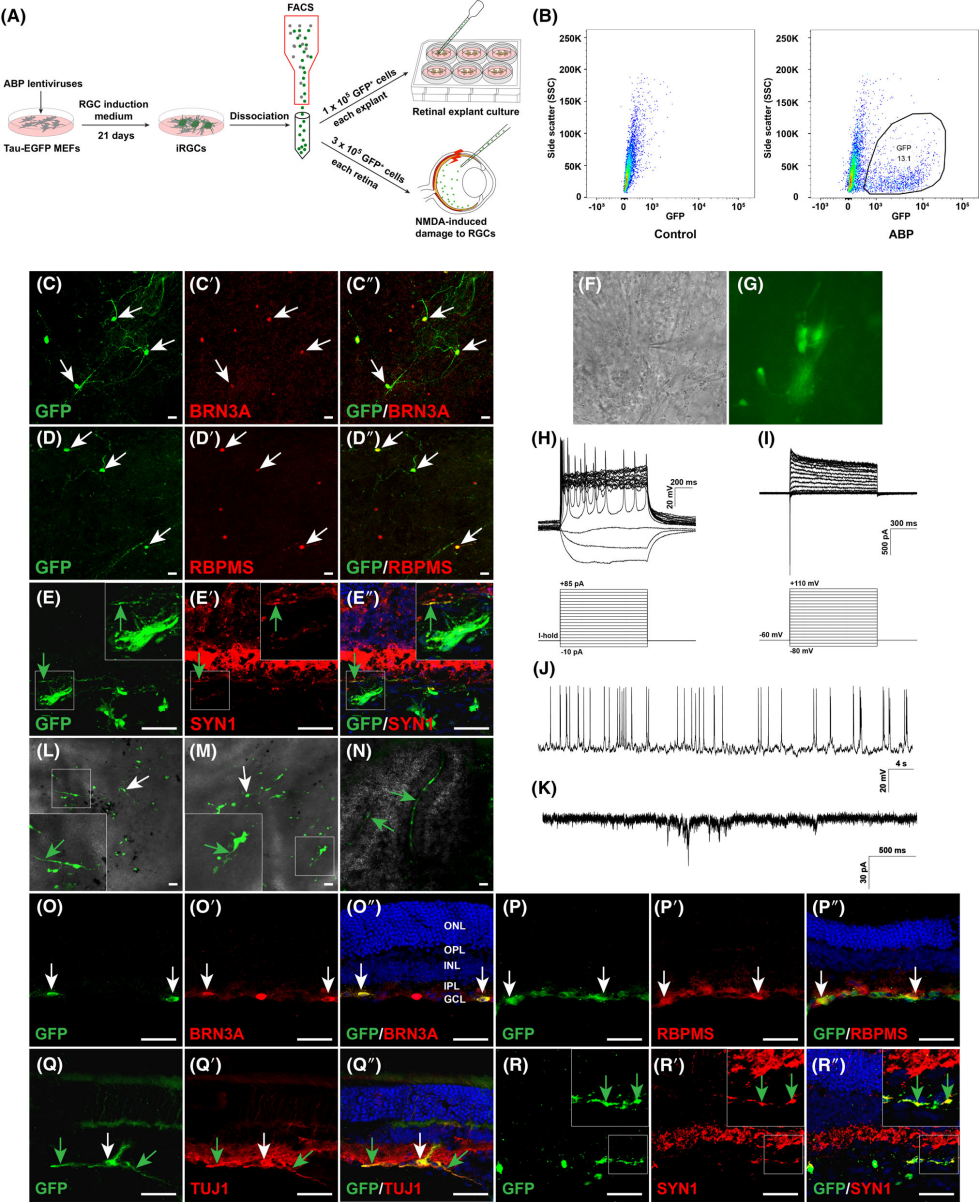
Survival and integration of ABP‐induced iRGCs in mouse retinal explants and NMDA‐damaged retinas. (A) Schematic illustration of the processes for isolation and transplantation of ABP‐induced GFP‐tagged iRGCs. (B) FACS analyses showing that GFP‐tagged iRGCs accounted for 13.1% of ABP‐transduced Tau‐EGFP MEFs. (C–C″, D–D″) Double‐immunostaining for GFP and RGC makers BRN3A or RBPMS in wholemount retinal explants on D10 after transplantation of ABP‐reprogrammed GFP‐tagged iRGCs. White arrows indicate colocalized cells. Scale bars, 40 μm. (E–E″) Double‐immunostaining for GFP and SYN1 in sections from retinal explants at D10 after transplantation of ABP‐reprogrammed GFP‐tagged iRGCs. Nuclei were stained with DAPI. Green arrows indicate colocalized iRGC fibres. Insets at the upper right corners are magnified from the corresponding outlined regions. Scale bars, 40 μm. (F,G) An ex vivo‐transplanted iRGC was chosen for patch‐clamp recording. (H) Current clamp recordings revealed multiple action potential (AP) responses (multiple‐spiking) of an ex vivo‐transplanted iRGC under current injection. I‐hold: a holding current injected to maintain the initial membrane potential at ~60 mV. (I) Voltage clamp recordings of the same neuron indicated fast activated and inactivated inward sodium currents as well as outward potassium currents. (J,K) Ex vivo‐transplanted iRGCs exhibited spontaneous APs (J) and sPSCs (K). (L–N) Merged bright‐field and fluorescent images of wholemount retinas showing the survival and morphology of ABP‐reprogrammed GFP‐tagged iRGCs 28 days after transplantation into NMDA‐damaged adult mouse retinas. Scale bars, 40 μm. White arrows point to cell bodies and green ones indicate iRGC fibres. Insets at the lower left corners are magnified from the corresponding outlined regions. (O–O″, P–P″, Q–Q″, R–R″) Double‐immunostaining for GFP and RGC markers BRN3A, RBPMS, TUJ1 or SYN1 in sections from retinas on D28 after transplantation of ABP‐reprogrammed GFP‐tagged iRGCs. Nuclei were stained with DAPI. White arrows indicate colocalized cells and green ones indicate colocalized iRGC fibres. GCL, ganglion cell layer; INL, inner nuclear layer; IPL, inner plexiform layer; ONL, outer nuclear layer; OPL, outer plexiform layer. Scale bars, 40 μm.

We infected Tau‐EGFP MEFs with ABP lentiviruses, and dissociated the cells followed by FACS on D21. The collected GFP‐tagged iRGCs were rapidly transplanted into either cultured mouse retinal explants or in vivo NMDA‐damaged mouse retinas (Figure 7A,B). We found that the number of host RGCs in the explants decreased greatly after 10 days of culture, consistent with previous findings that mouse RGCs cultured in vitro die rapidly within ~4–7 days.^36^ Meanwhile, the transplanted GFP‐tagged iRGCs not only integrated well into the GCL of the explants, but also expressed neuronal maker TUJ1 and RGC markers BRN3A and RBPMS, and exhibited a morphology similar to that of endogenous RGCs (Figures 7C–C″,D–D″ and S9A–A″,B–B″,C–C″). In addition, they were capable of regrowing nerve fibres and expressed the synaptic protein Synapsin (Figure 7C–C″,D–D″,E–E″). Next, we performed whole‐cell patch‐clamp recordings of GFP‐expressing iRGCs with neuronal morphology in explants (Figure 7F,G). The majority of recorded iRGCs (9 of 11) had typical sodium and potassium currents and fired multiple APs when evoked, suggesting that they were functionally mature (Figure 7H,I). Moreover, some neurons (3 of 11) fired spontaneous APs, and consistent with the Synapsin immunoreactivity, sPSCs were also detected (3 of 14) (Figure 7J,K), indicating the formation of functional synapses between iRGCs and host neurons in retinal explants. We also found that in NMDA‐damaged retinas, GFP‐tagged iRGCs could still survive and regrow dendrites and axons in the damaged host retina 28 days after transplantation (Figure 7L–N). Immunofluorescence staining showed that the majority of surviving GFP‐tagged iRGCs integrated into the GCL and expressed neuronal marker TUJ1 and RGC markers BRN3A, RBPMS, HUC/D or THY1.2 (Figures 7O–O″,P–P″,Q–Q‴ and S9D–D″,E–E″). In agreement with the observation in retinal explants, some GFP‐tagged iRGCs also regrew nerve fibres and expressed the synaptic protein Synapsin after integration into the host retina (Figures 7Q–Q″,R–R″ and S9F–F″). These results suggest that ABP‐induced RGCs are able to survive, integrate into the GCL of the retina, and regrow dendrites and axons.

## 3 DISCUSSION

At present, the regulation mechanism of RGC development in vivo is relatively clear, and TFs play important roles in the fate determination, specification and differentiation of RGCs.^24^ In recent years, many RGC regulatory TFs such as BRN3A/3B (POU4F1/2),^27^, ^37^ MATH5 (ATOH7),^38^ SOX4/11,^39^ EBF1–4,^40^ ISL1,^28^ DLX1/2^41^ and PAX6,^42^, ^43^ among others, have been identified. Direct cell reprogramming in vitro mimics much of the in vivo developmental regulation of reprogrammed cells. However, neither single RGC TFs nor their different combinations were able to reprogram MEFs into cells expressing RGC‐specific markers, and RGC‐like neurons expressing BRN3A did not appear until the neuronal transcription factor ASCL1 was added and combined with ISL1 or BRN3B (Figures 1 and S1).^21^ These suggest that when BRN3B and ISL1 is combined with ASCL1, the ‘pioneer factor’ ASCL1 exhibits ‘on‐target’ capability in promoting the effective induction of MEFs into iSG neurons and/or iRGCs, which is similar to its role in BAM (BRN2 + ASCL1 + MYT1L) for inducing iNs (Figure 1),^44^ and it appears that ISL1 and BRN3B play a more critical role than other RGC TFs in inducing MEFs to transdifferentiate into RGC‐like neurons in vitro. Previous studies have demonstrated that two TFs, BRN3B and ISL1, are together sufficient to specify the RGC fate in the mouse retina.^28^, ^45^ However, our recent study showed that only about 12% of the neurons induced by ABI through reprogramming from MEFs were RGC‐like neurons, and the rest were iSG neurons.^21^ We speculate that the main reason is that ISL1 not only regulates RGC development, but also plays a crucial role in sensory neuron development, which has been confirmed in previous studies.^46^, ^47^ In addition, the induction medium used in this work was modified from the previously published neuron induction medium, which may be more suitable for induction of iSG neurons.^48^

In this study, our new combination of ASCL1 and BRN3B with PAX6 instead of ISL1 was significantly more efficient than ABI in reprogramming iRGCs. We provided evidence to show that ABP‐induced iNs were mostly iRGCs rather than iSG neurons (Figures 1 and S8), suggesting that the combination of PAX6 and BRN3B was able to direct ASCL1‐initiated neuron transdifferentiation toward an RGC differentiation path. This also highlights the critical role of PAX6 in RGC differentiation as reported previously.^42^, ^43^, ^49^ Moreover, we found that cells expressing RGC‐specific markers (BRN3A, NEFM and SNCG) appeared as early as D6 after ABP transduction of MEFs, indicating that ABP induced iRGCs rapidly (Figures 5 and S5). However, it took at least 3 weeks to differentiate RGCs from stem cells (ESCs/iPSCs) using previously established methods.^10^, ^13^, ^50^ This may be due to the need for gradual inhibition of stem cell totipotency and slow differentiation toward the target cells, often through an intermediate state. In contrast, our EdU pulse‐labelling experiment showed that ABP directly induced MEFs to produce iRGCs without undergoing an intermediate precursor cell stage (Figure S6). In addition, the only medium used for the ABP‐induction process was optimized from the ABI‐induction medium, mainly with the addition of Dkk1 and Noggin, which have been shown in previous publications to be involved in the regulation of developmentally relevant signalling pathways in the retina.^12^, ^51^, ^52^ However, previous studies have shown that at least two differentiation media are needed to derive RGCs from stem cells.^10^, ^13^, ^50^ The use of multiple media may prolong the cell adaptation period to microenvironments and increase the complexity of the experimental procedure.

One of the main goals in RGC research is to obtain donor cells for transplantation and cell replacement therapy in conditions such as glaucoma and other optic neuropathies. In this study, we assessed the potential of ABP‐induced RGCs as donor cells through transplantation into mouse retinal explants and NMDA‐damaged retinas. Our results demonstrated the good survival and integration ability of transplanted iRGCs (Figures 7 and S9). Specifically, while most endogenous RGCs in retinal explants only survived for 4–7 days in vitro,^36^ iRGCs not only survived for 10 days after transplantation onto explants, but also could regrow dendrites and axons, and exhibit RGC‐like morphology. In addition, transplanted iRGCs showed positive Synapsin immunoreactivity and electrophysiological properties such as sAPs and sPSCs, suggesting that iRGCs form synaptic connections with host explant cells. More importantly, 1 month after intravitreal transplantation of iRGCs into NMDA‐damaged retinas, they still survived and integrated into the GCL, and some iRGCs regrew dendrites and axons, which was similar to the transplantation of primary RGCs into rat retinas.^5^ Thus, iRGCs directly reprogrammed from MEFs through ABP induction are a good potential cell source for cell transplantation therapy of glaucoma and other optic neuropathies.

In summary, in a screen for TFs capable of inducing iRGCs, we have identified a triple‐factor combination ABP that is able to quickly and efficiently reprogram MEFs into iRGCs. Through various approaches including immunofluorescence staining, qRT‐PCR, whole‐cell patch‐clamp recording, and bulk RNA‐seq and scRNA‐seq, we were able to demonstrate that ABP‐induced iRGCs exhibited molecular, cellular and electrophysiological properties of native RGCs. Furthermore, following transplantation, ABP‐induced iRGCs survived, successfully integrated into mouse retinal explants and NMDA‐damaged retinas, and regrew dendrites and axons. Overall, the approach described here can enable highly efficient and rapid generation of iRGCs, and its simplicity will benefit translational studies on RGCs. Moreover, this work may also provide new insights for efficient regeneration of RGCs in vivo, especially for inducing retinal Müller glia to regenerate RGCs.

## 4 MATERIALS AND METHODS

### 4.1 Animals

All experiments on rodents were performed according to the Institutional Animal Care and Use Committee (IACUC) standards and approved by Sun Yat‐sen University and Zhongshan Ophthalmic Center. Adult C57BL/6 mice were purchased from The Vital River Laboratories (China). The Tau‐EGFP mice were purchased from The Jackson Laboratory (#029219).^53^ The *Brn3b*‐GFP mice were generated in our own laboratory and will be published elsewhere.

### 4.2 Construction of viral plasmids and preparation of lentiviruses

The full‐length open reading frames (ORFs) of *Ascl1*, *Brn3b*, *Pax6*, *Dlx1*, *Ebf1* or *Isl1* were subcloned into the multiple cloning site (MCS) of the FUW‐TetO vector, a lentiviral vector containing the tetracycline operator (TetO), a minimal cytomegalovirus (CMV) promoter and the FUW backbone.^54^ The FUW‐tetON‐eGFP plasmid was purchased from Addgene (no. 115495). Lentiviruses were prepared as described previously^55^ using the Hieff Trans® Liposomal Transfection Reagent (Yeasen, China) according to the manufacturer's instruction.

### 4.3 Preparation of MEFs

The MEFs were prepared as described previously.^18^ In brief, E14.5–E16.5 C57BL/6 or Tau‐EGFP mouse embryos were quickly collected and placed in a cell culture dish containing pre‐cooled Hanks' balanced salt solution (HBSS) (HyClone). After rinse with cold HBSS, the head, tail, limbs, spinal cord and viscera were removed under a stereo microscope (Leica). The skin tissue was carefully separated with fine forceps (FST), thoroughly minced into small pieces using surgical scissors (FST), and digested with 0.25% trypsin‐ethylenediaminetetraacetic acid (EDTA) (Gibco) at 37°C for 15 min. The digestion was terminated with the addition of the MEF medium [Dulbecco's modified Eagle's medium (DMEM) (Gibco) supplemented with 10% foetal bovine serum (FBS) (Gibco), 1× Penicillin/Streptomycin (Pen/Strep) (Gibco), and 1× minimum essential medium (MEM) non‐essential amino acids (NEAA) (Gibco)], and the digested tissue was triturated to dissociate cells by carefully pipetting up and down several times. The cell suspension was centrifuged at 225*g* for 5 min and resuspended with fresh MEF medium. Cells derived from two embryos were pooled into one 75‐cm^2^ flask (Corning), cultured in a humidified CO_2_ incubator (Thermo Fisher) at 37°C, and expanded by no more than two passages.

### 4.4 Fibroblast reprogramming

To induce iRGCs from MEFs, 3 × 10^4^ MEFs (at passage 2) were cultured in 500 μL MEF medium in a well of a 24‐well plate (Corning) containing a 12‐mm cover glass (VWR) precoated with 1.25% Matrigel (Corning) in MEM (Gibco). They were infected the next day with 500 μL mixture of lentiviruses and fresh MEF medium in the presence of hexadimethrine bromide (10 μg/mL) (Sigma). After 16 h of infection, the lentivirus and medium mixture was removed. The cells were induced for 2 days in MEF medium supplemented with basic fibroblast growth factor (bFGF, 10 ng/mL) (PeproTech) and doxycycline (2 μg/mL) (Sigma), and for another 12 days in RGC induction medium [DMEM/F12 (1:1) (Gibco) supplemented with 1× Pen/Strep, 1× B27 (Gibco), bFGF (10 ng/mL), insulin‐like growth factor 1 (IGF‐1, 100 ng/mL) (PeproTech), brain‐derived neurotrophic factor (BDNF, 10 ng/mL) (PeproTech), glial cell derived neurotrophic factor (GDNF, 10 ng/mL) (PeproTech), β‐nerve growth factor (β‐NGF, 10 ng/mL) (PeproTech), neurotrophin‐3 (NT‐3, 10 ng/mL) (PeproTech), Dkk‐1 (20 ng/mL) (R&D Systems), Noggin (20 ng/mL) (R&D Systems), and doxycycline (2 μg/mL)]. The medium was half‐changed every other day, and doxycycline was removed on D14.

### 4.5 Immunofluorescence staining

Cells grown on cover glasses were fixed with 4% (w/v) paraformaldehyde (PFA) (Sigma) in phosphate buffered saline (PBS) for 15 min at room temperature. After washing with PBS for three times, the cells were blocked with cell blocking buffer [5% (w/v) bovine serum albumin (BSA) (Sigma), 1% (v/v) normal donkey serum (Jackson ImmunoResearch), 1% (v/v) normal goat serum (Jackson ImmunoResearch) and 0.2% (v/v) Triton X‐100 (Sigma) in PBS] for 1 h at room temperature and incubated at 4°C overnight with primary antibodies diluted in cell blocking buffer. After washing with PBS for three times, the cells were incubated with secondary antibodies and 4′,6‐diamidino‐2‐phenylindole (DAPI) (Invitrogen) diluted in cell blocking buffer for 1 h at room temperature.

Tissue processing was carried out as described previously.^56^ Retinas or retinal explants were embedded in the OCT compound (Sakura). The frozen tissue‐OCT blocks were cut into 15 μm sections using a cryostat (Leica) and the sections were mounted on adhesive microscope slides (Citotest, China). Following three rinses with 0.1% Triton X‐100 in PBS (0.1% PBST) (for sections) or 0.3% Triton X‐100 in PBS (0.3% PBST) (for wholemounts), retinal sections and wholemounts were blocked with section blocking buffer (10% donkey serum in 0.1% PBST), or wholemount blocking buffer (10% donkey serum in 0.3% PBST) for 1 h at room temperature, and incubated with primary antibodies diluted in section blocking buffer at 4°C overnight, or in wholemount blocking buffer at 4°C for 48 h on an orbital shaker. After three rinses with 0.1% PBST (for sections) or 0.3% PBST (for wholemounts), they were incubated with secondary antibodies and DAPI diluted in section blocking buffer or wholemount blocking buffer for 1 h at room temperature. Primary antibodies used were listed in Table S1. Images were captured by the LSM700 confocal system (Carl Zeiss).

### 4.6 Quantitative real‐time PCR and EdU labelling

Total RNA was extracted from cultured cells using NucleoZOL (Macherey‐Nagel) according to the manufacturer's instruction. cDNA was synthesized using the HiScript III RT SuperMix for qPCR (Vazyme, China). Quantitative real‐time PCR (qRT‐PCR) was performed using the KAPA SYBR® FAST qPCR Master Mix (2×) Kit and qTower^3^G Real‐Time PCR system (AnalytikJena). The data were analysed using the 2^−ΔΔct^ calculation method. The qRT‐PCR primers used are listed in Table S2.

EdU labelling was performed as described previously using the Click‐iT® EdU Imaging Kit (Invitrogen).^21^ MEFs were labelled with 10 μM EdU for 19 days starting from D2, or for 24 h starting from D20. EdU staining was carried out according to the manufacturer's instruction.

### 4.7 Time‐lapse recording

MEFs (5 × 10^4^) derived from C57BL/6 embryos were induced for 10 days by infection with the ABP (ASCL1 + BRN3B + PAX6) lentiviruses or ASCL1 lentivirus in a well of a 12‐well plate (Corning) precoated with 1.25% Matrigel. The plate was placed into the cell incubator coupled with the JuLI Stage real‐time cell history recorder (NanoEntek Inc) for 50 h. A series of pictures were taken from each well of the 12‐well plate in a period of 50 h under the control of the JuLI EDIT software, and edited in a continuous mode to generate a movie.

### 4.8 Fluorescence activated cell sorting

For cells reprogrammed from Tau‐EGFP MEFs, fluorescence activated cell sorting (FACS) was used to isolate GFP‐tagged iRGCs for further experiments. Cells were dissociated using Accutase (Innovative Cell Technologies). After enzyme inactivation, the cell suspension was centrifuged at 225*g* for 5 min. The precipitated cells were resuspended in Dulbecco's phosphate buffered saline (DPBS) with 2% FBS and 1 mM EDTA, and passed through a 40‐μm cell strainer (Falcon) into a 5‐mL round bottom tube (Falcon). FACS was carried out on a BD FACSAria™ Fusion cell sorter (BD Biosciences), gated for a high level of GFP expression.

### 4.9 Bulk RNA‐seq and scRNA‐seq analyses

Bulk RNA‐seq analysis was carried out as described previously.^18^, ^21^ For bulk RNA‐seq experiment at each time point, cells grown in two wells of a 24‐well plate were used for each sample. Total RNA was extracted from these cells for library preparation. Ribosomal RNA was depleted prior to RNA‐seq library preparation. The obtained sequence reads were trimmed and mapped to the mm10 mouse reference genome.

For single‐cell RNA sequencing (scRNA‐seq) analysis, cells derived from Tau‐EGFP MEFs (sorted or unsorted) on D21 were used. Single‐cell library construction, sequencing, Cell Ranger processing and Seurat analysis were performed as described previously,^21^, ^57^ and clusterProfiler was applied to perform the GO pathway enrichment analyses.^58^

### 4.10 Retinal explant and cell transplantation

Retinal explant culture was conducted as described previously.^59^ In brief, the eyeballs were isolated from P7 C57BL/6 pups, from which retinal cups were made by carefully removing the sclera, choroid and lens. Four incisions were made from the margin of the retinal cup half way toward the bottom at the 0, 3, 6 and 9 o'clock positions. Retinas were transferred using a Pasteur pipette onto Millicell cell culture inserts (Millipore) with the GCL facing up (three explants per insert). The inserts were placed into a 6‐well plate (Corning) with 1 mL explant culture medium [DMEM/F12 (1:1) (Gibco) supplemented with 1× Pen/Strep, 15% FBS (Gibco), 5 μM Forskolin (Sigma) and 1× insulin/transferrin/selenium (Invitrogen)] in each well. The plate was transferred into a humidified cell culture incubator (37°C, 5% CO_2_).

Cell transplantation was carried out 12 h after the explants were prepared. Cells induced from Tau‐EGFP MEFs were collected and sorted on D21. iRGCs were resuspended in explant culture medium and transplanted directly over the centre of the explants (100,000 cells per explant). The medium was half‐changed every other day. After 10 days of transplantation, explants were collected for immunofluorescence staining or electrophysiological recordings.

### 4.11 NMDA‐induced retinal damage and cell transplantation

Three‐month‐old C57BL/6 mice were anaesthetised with an intraperitoneal injection of pentobarbital sodium (Sigma), and one drop of 0.5% tetracaine hydrochloride (Zhongshan Ophthalmic Center) was administered on the eyeball for local anaesthesia. Tropicamide (Santen, China) was used to dilate the pupils, and 150 μmol NMDA (1.5 μL) (Sigma) was injected intravitreally into the eye.

Cell transplantation was performed 7 days after NMDA injection. Cells induced from Tau‐EGFP MEFs were collected and sorted on D21. iRGCs were resuspended in PBS and injected intravitreally into the eye (300,000 cells, 1.5 μL per retina). After 28 days of transplantation, mice were euthanized and retinas were collected for subsequent experiments.

### 4.12 Electrophysiology

All chemicals used in this section were purchased from Sigma unless specified otherwise. Whole‐cell patch clamp recordings were performed with a MultiClamp 700B amplifier (Molecular Devices) connected to a Digidata 1550B interface (Molecular Devices). Data were acquired and analysed with the pClamp 11.2 software package.

One 12‐mm cover glass with adherent cells was placed in the recording chamber (Warner Instruments) on the fixed stage of an upright microscope (Olympus) equipped with a 40× water‐immersion objective, and cells were visually identified through a monitor coupled with a digital CMOS camera (Hamamatsu). The recording chamber was continuously perfused at a rate of 1.5–2 mL/min controlled by a peristaltic pump (LongerPump, China) with oxygenated Ringer's solution consisting of (in mM): 125 NaCl, 2.5 KCl, 1 MgSO_4_, 2 CaCl_2_, 1.25 NaH_2_PO_4_, 26 NaHCO_3_, and 20 d‐glucose. The osmolarity was adjusted to 290–300 mOsm/L with sucrose.

For retinal explants, the membrane of the Millicell insert was carefully cut around the explant. For *Brn3b*‐GFP mouse retinas, after euthanasia of the animal, the dissection was carried out quickly in oxygenated (95% O_2_, 5% CO_2_) Ames' medium supplemented with 1.9 g/L NaHCO_3_ (osmolarity 290–300 mOsm/L), and four perpendicular incisions were made from the margin of the dissected retinal cup half way toward the bottom. The retinal explant or the retina was mounted flat with GCL facing up in the recording chamber. A slice anchor (Warner Instruments) was put on the explant or the retina to secure it. GFP was excited using the Lambda DG‐4 light source system (Sutter Instrument). The recording chamber was heated to 34°C by the TC‐344C temperature controller (Warner Instruments), and continuously perfused at a rate of 1.5–2 mL/min with oxygenated Ames' medium supplemented with 1.9 g/L NaHCO_3_ (osmolarity 290–300 mOsm/L).

Borosilicate glass (WPI) electrodes were pulled to a resistance of 5–7 MΩ using a P‐1000 micropipette puller (Sutter Instrument). For action potential (AP) and whole‐cell current recordings, the micropipettes were filled with K‐gluconate internal solution consisting of (in mM): 126 K‐gluconate, 4 KCl, 0.05 ethylene glycol‐bis(2‐aminoethylether)‐N,N,N′,N′‐tetraacetic acid (EGTA), 10 4‐(2‐hydroxyethyl)piperazine‐1‐ethanesulfonic acid (HEPES), 4 adenosine 5′‐triphosphate magnesium (Mg‐ATP), 0.3 guanosine 5′‐triphosphate sodium (Na‐GTP), and 10 Na_2_‐phosphocreatine. The pH was adjusted to ~7.2 with 0.1 M KOH, and osmolarity to 270–290 mOsm/L with sucrose. Spontaneous APs were recorded in current clamp mode, first with no current injection, and then with a holding current injection to maintain a membrane potential of ~65 mV (cultured cells) or ~60 mV (explants or retinas). Stepwise current evoked APs were recorded in current clamp mode, with a holding current (I‐hold) injected to maintain the initial membrane potential at ~65 mV (cultured cells) or ~60 mV (explants or retinas). Current pulses were applied from I‐hold −10 to +85 pA at 5 pA intervals. Whole‐cell currents were recorded in voltage clamp mode with a holding potential of −70 mV (cultured cells) or −60 mV (explants or retinas), and voltage steps ranging from −90 to +100 mV (cultured cells) or −80 to +110 mV (explants or retina) were delivered at 10 mV increments. To inhibit voltage gated sodium channels, tetrodotoxin (TTX) (Aladdin, China) was added to the Ringer's solution to a final concentration of 2 μM. For spontaneous post synaptic current (sPSC) recording, the micropipettes were filled with CsCl internal solution consisting of (in mM): 135 CsCl, 5 EGTA, 10 HEPES, 4 Mg‐ATP, 0.3 Na‐GTP and 10 Na_2_‐phosphocreatine. The pH was adjusted to ~7.2 with 0.1 M CsOH, and osmolarity to 270–290 mOsm/L with sucrose. sPSCs were recorded in voltage clamp mode, with the cells' membrane potential held at −70 mV (cultured cells) or −60 mV (explants or retinas).

### 4.13 Statistics

Statistical analyses were performed using the GraphPad Prism 9 and Microsoft Excel software. The results were expressed as mean ± SD for experiments conducted at least in triplicates. Unpaired two‐tailed Student's *t* test or one‐way ANOVA with Bonferroni's correction were used to assess differences between two groups, and a value of *p* < 0.05 was considered statistically significant.

## AUTHOR CONTRIBUTIONS

Mengqing Xiang and Dongchang Xiao conceived and designed the research. Zihui Xu, Dongchang Xiao and Mengqing Xiang performed the experiments and analysed the data. Yanan Guo and Kangjian Xiang took care of animal breeding and maintenance. Zihui Xu, Dongchang Xiao and Mengqing Xiang interpreted the data and wrote the manuscript. All authors contributed to critical reading of the manuscript.

## CONFLICT OF INTEREST STATEMENT

The authors have declared that no conflict of interest exists.

## Supporting information


**Data S1.** Supplementary Information.

**Table S3. (Microsoft Excel format).** List of differentially expressed genes (fold change ≥2 and *p*‐value <0.05) between ABP‐transduced and GFP‐transduced MEFs (D21) as determined by bulk RNA‐seq analysis.

**Videos S1.** MEFs were induced by ABP (ASCL1, BRN3B and PAX6) for 10 days, and then recorded by long‐term time‐lapse microscopy for 50 h. The recording results showed that ABP‐induced neurons would self‐organize into neuronal clusters.

**Videos S2.** MEFs were induced by A (ASCL1) for 10 days, and then recorded by long‐term time‐lapse microscopy for 50 h. The recording results showed that no self‐organization was observed for ASCL1‐induced neurons.

## Data Availability

The bulk RNA‐seq and scRNA‐seq data have been deposited in the NCBI Sequence Read Archive (SRA) database and are publicly available as of the date of publication. The accession number is PRJNA1002073. Other data that support the findings of this study are available from the corresponding authors upon reasonable request.
