# Supplementary material for: Rapid and efficient generation of a transplantable population of functional retinal ganglion cells from fibroblasts
Source: Cell Prolif. 2023 Sep 23;57(2):e13550. doi: 10.1111/cpr.13550 (PMC10849786; doi:10.1111/cpr.13550)
Supplement: Supplementary file 1 — Data S1. Supplementary Information. [file CPR-57-e13550-s004.pdf]

Rapid and efficient generation of a transplantable population of functional retinal ganglion cells from fibroblasts

Zihui Xu[1], Yanan Guo[1], Kangjian Xiang[1], Dongchang Xiao[1*], Mengqing Xiang[1,2*]

[1]State Key Laboratory of Ophthalmology, Zhongshan Ophthalmic Center, Sun Yat-sen University, Guangdong Provincial Key Laboratory of Ophthalmology and Visual Science, Guangzhou 510060, China
[2]Guangdong Provincial Key Laboratory of Brain Function and Disease, Zhongshan School of Medicine, Sun Yat-sen University, Guangzhou, 510080, China

Supplementary Figures S1–9 and Tables S1, S2
Legend for Table S3
Legends for Videos S1, S2

A

TF / Marker	ASCL1 (A)	BRN3B (B)	DLX1 (D)	EBF1 (E)	ISL1 (I)	PAX6 (P)
Tuj1	P	N	N	N	N	N
Brn3a	N	N	N	N	N	N

B

TF / Marker	AB	AD	AE	AI	AP
Tuj1	P	P	P	P	P
Brn3a	P	N	N	P	N

C

TF / Marker	ABI	ABD	ABE	ABP	AID	AIE	API
Tuj1	P	P	P	P	P	P	P
Brn3a	P	P	P	P	P	P	P

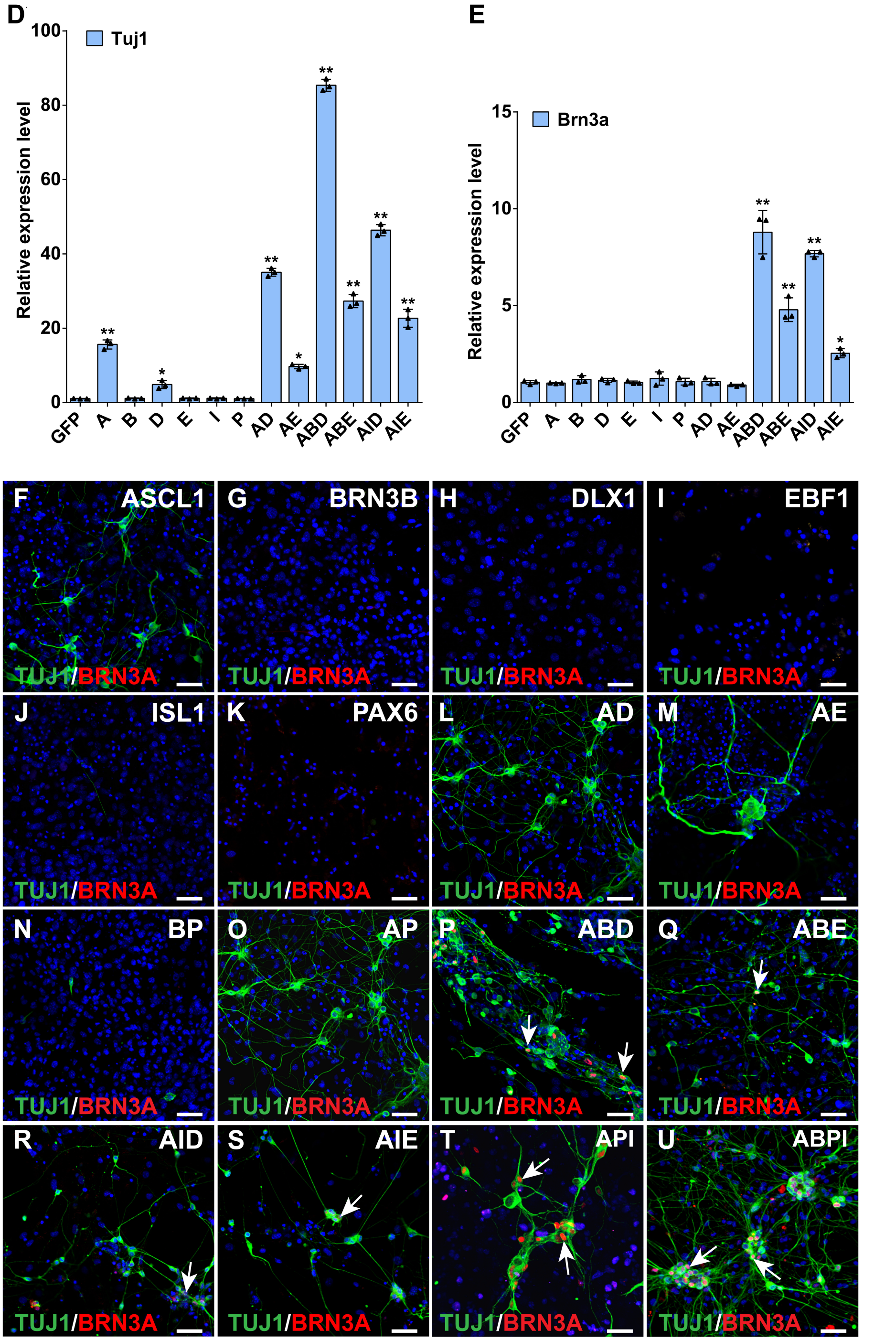

Figure S1. Screening of TFs for iRGC induction. (A) Positive (P) or negative (N) immunostaining results of TUJ1 and BRN3A in MEFs infected with the indicated single TF lentiviruses (A, ASCL1; B, BRN3B; D, DLX1; E, EBF1; I, ISL1; P, PAX6). Only ASCL1 could induce TUJ1-positive neurons. (B) Positive or negative immunostaining results of TUJ1 and BRN3A in MEFs co-infected with both ASCL1 and the other indicated TFs. Only AI- and AB-reprogrammed neurons co-expressed TUJ1 and BRN3A. (C) Positive or negative immunostaining results of TUJ1 and BRN3A in MEFs infected with the indicated triple-TF lentiviruses. All the triple-TF combinations were able to induce TUJ1 and BRN3A co-expressing neurons. (D, E) qRT-PCR analyses showing relative expression levels of *Tuj1* and *Brn3a* in MEFs infected with the indicated lentiviruses on D21 post induction. Data are presented as mean ± SD (n = 3). Asterisks indicate significance in one-way ANOVA test: $*p < 0.001$, $**p < 0.0001$. (F–U) Double-immunostaining for TUJ1 and BRN3A in MEFs on D21 after infection with the indicated lentiviruses. Nuclei were stained with DAPI. Arrows indicate colocalized cells. Scale bars, 40 µm.

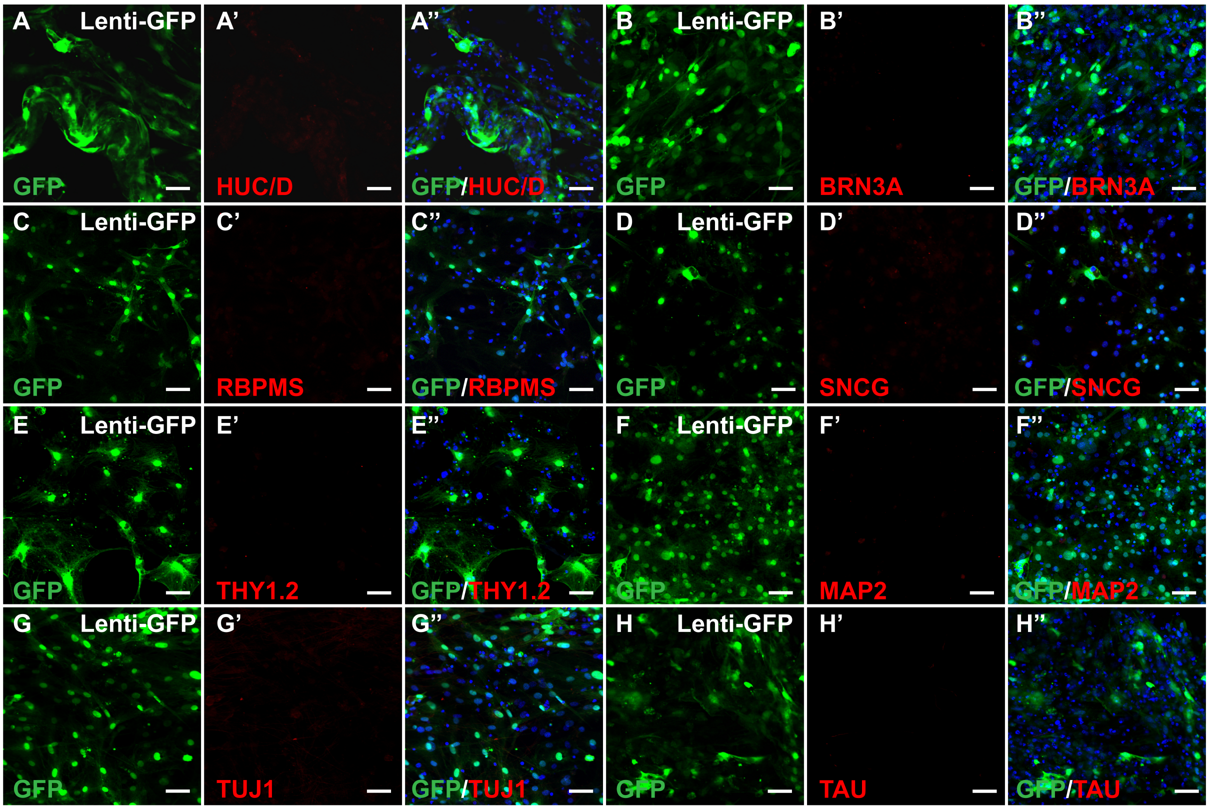

Figure S2. Negative immunostaining results of RGC and neuron markers in GFP-transduced MEFs. RGC markers include HUC/D, BRN3A, RBPMS, SNCG, and THY1.2. Neuron markers include TUJ1, MAP2 and TAU. Nuclei were stained with DAPI. Scale bars, 40 µm.

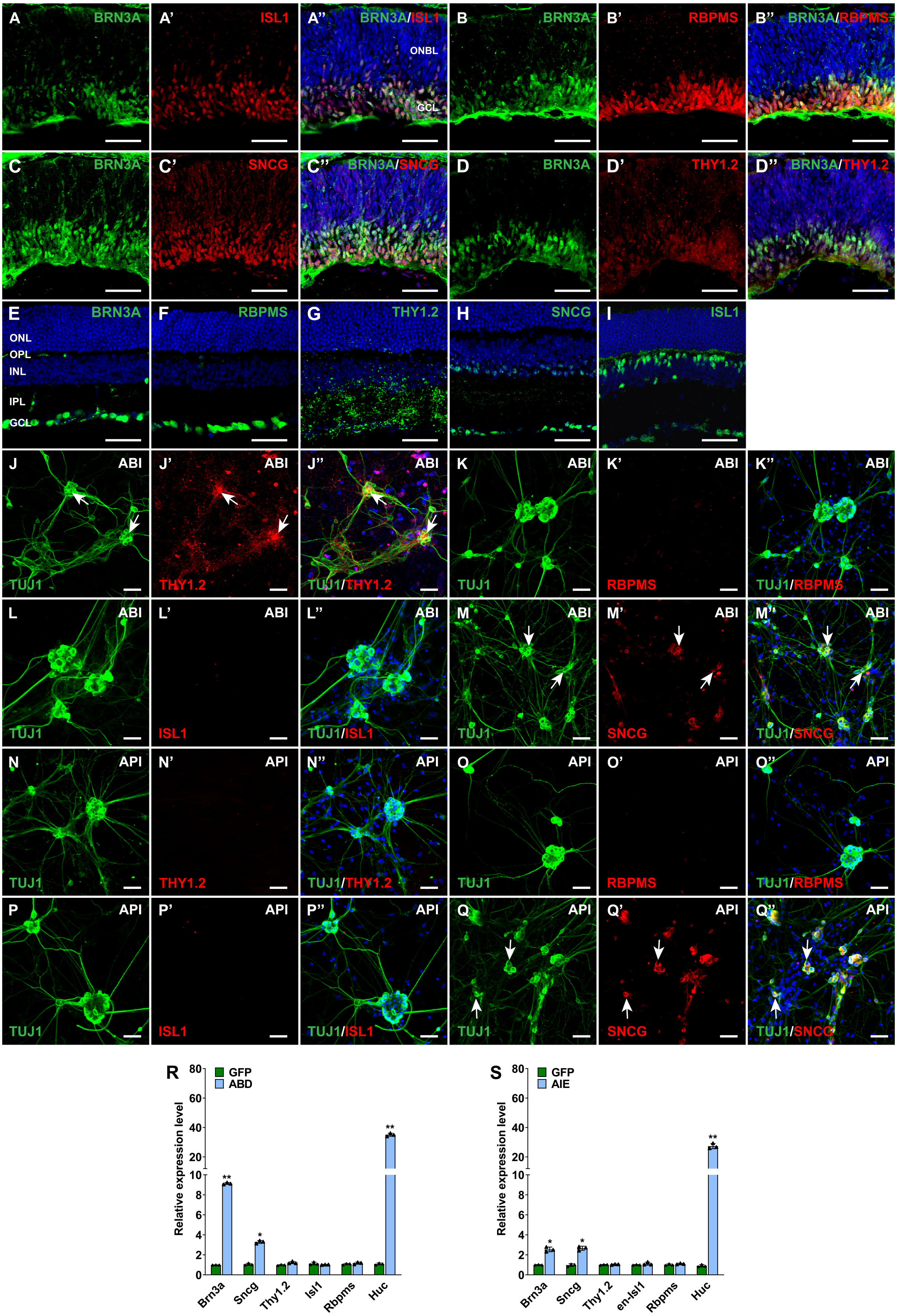

Figure S3. Detection of a few commonly used RGC markers in the mouse retina, and

in ABI-, API-, ABD-, and AIE-transduced MEFs. (**A–A”, B–B”, C–C”, D–D”**) Immunostaining results showing that E14.5 mouse retinal sections were co-labeled for both BRN3A and the other indicated RGC markers (ISL1, RBPMS, SNCG, or THY1.2). Nuclei were stained with DAPI. GCL, ganglion cell layer; ONBL, outer neuroblastic layer. Scale bars, 40 µm. (E–I) Adult mouse retinal sections were immunostained with the indicated antibodies. Nuclei were stained with DAPI. GCL, ganglion cell layer; INL, inner nuclear layer; IPL, inner plexiform layer; ONL, outer nuclear layer; OPL, outer plexiform layer. Scale bars, 40 µm. (J–J”, K–K”, L–L”, M–M”, N–N”, O–O”, P–P”, Q–Q”) ABI- and API-transduced MEFs were double-immunostained for TUJ1 and RGC makers THY1.2, RBPMS, ISL1, or SNCG. Nuclei were stained with DAPI. Scale bars, 40 µm. Arrows indicate colocalized cells. (R, S) qRT-PCR analyses showing relative expression levels of the indicated RGC maker genes in GFP-, ABD- or AIE-transduced MEFs. The amplificon of endogenous *Isl1* (*en-Isl1*) primers comes from the 3' end of *Isl1* mRNA. Data are presented as mean ± SD (n = 3). Asterisks indicate significance in unpaired two-tailed Student′s t test: *p < 0.05, **p < 0.0001.

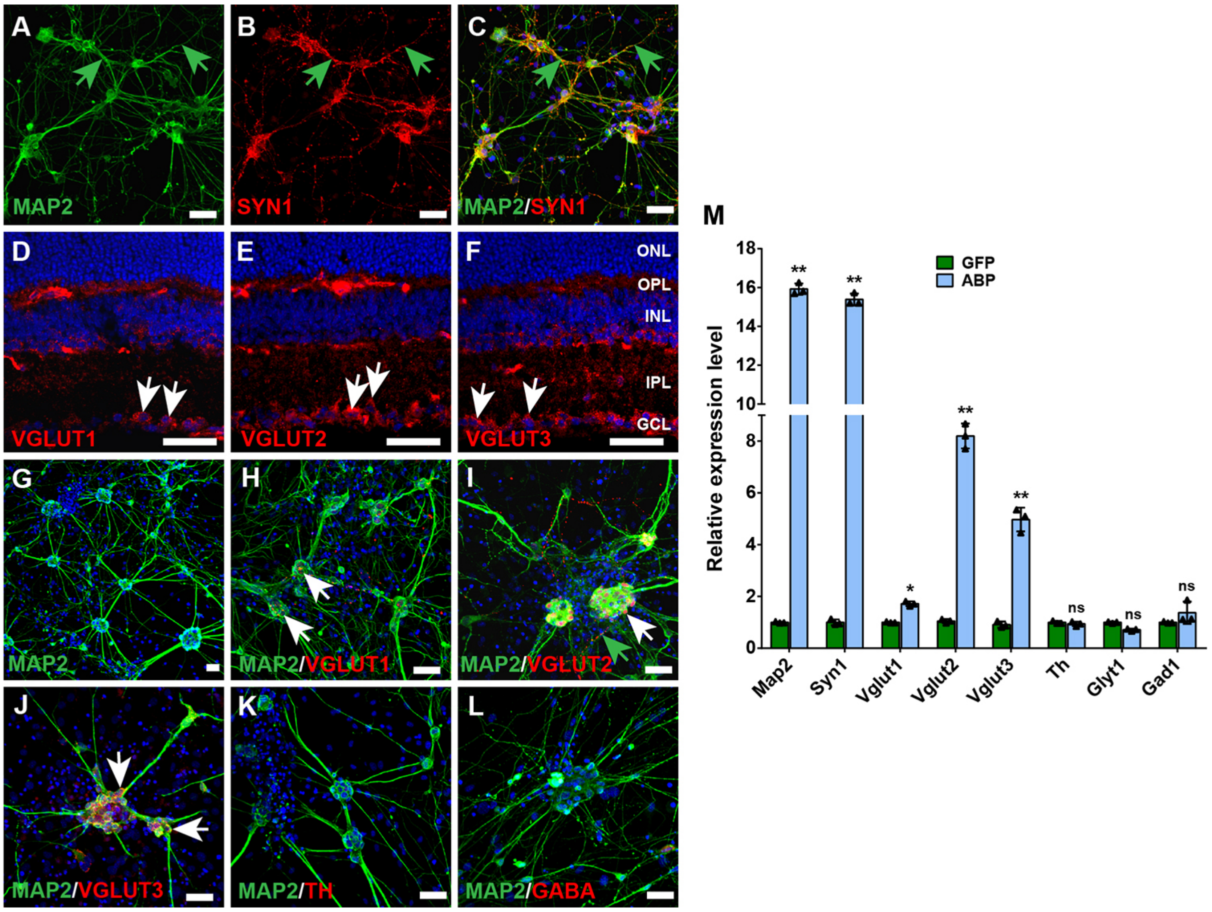

Figure S4. ABP-induced iRGCs are glutamatergic neurons. (A–C) ABP-induced iRGCs were double-immunostained for mature neuronal markers MAP2 and SYN1 (Synapsin1). Nuclei were stained with DAPI. Green arrows point to colocalized neuron fibers. Scale bars, 40 µm. (D–F) Adult mouse retinal sections were immunostained with the indicated antibodies against the three types of vesicular glutamate transporters (VGLUT1–3). Nuclei were stained with DAPI. White arrows indicate immunoreactive RGCs. GCL, ganglion cell layer; INL, inner nuclear layer; IPL, inner plexiform layer; ONL, outer nuclear layer; OPL, outer plexiform layer. Scale bars, 40 µm. (G–L) ABP-induced iRGCs were immunostained with the indicated antibodies (MAP2, VGLUT1–3, TH, and GABA). Nuclei were stained with DAPI. White arrows indicate colocalized cells and the green one points to a colocalized neuron fiber. Scale bars, 40 µm. (M) qRT-PCR analysis showing relative expression levels of the indicated mature and subtype-specific neuron marker genes in GFP- and ABP-transduced MEFs. Data are presented as mean ± SD (n = 3). Asterisks indicate significance in unpaired two-tailed Student′s t test: *$p < 0.05$, **$p < 0.0001$. ns, no significance.

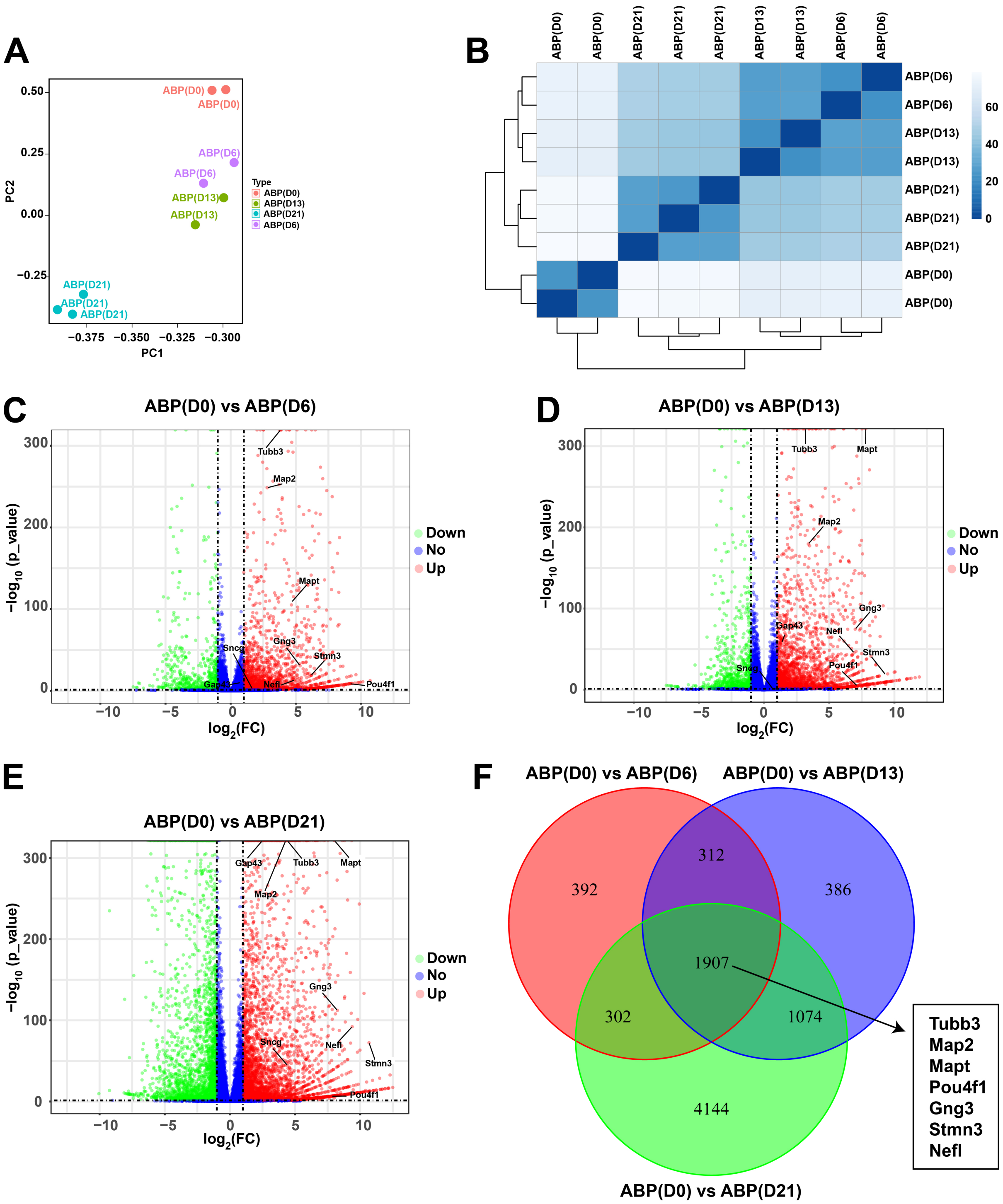

Figure S5. Global gene expression profiles determined by bulk RNA-seq analysis at four time points during the iRGC induction process. (A) Principal component analysis (PCA) of the gene expression profiles of the indicated samples. ABP(D0-21): ABP-transduced MEFs on day 0-21. (B) Heatmap showing the correlations among the indicated samples. (C–E) Volcano plots showing gene expression changes between D0 and 6 (C), D0 and 13 (D), and D0 and 21 (E) after ABP lentivirus infection. Some representative neuron

marker genes (*Tubb3*, *Map2*, *Mapt*) and RGC marker genes (*Pou4f1*, *Gng3*, *Stmn3*, *Nefl*, *Gap43*, *Sncg*) are highlighted. (F) Venn diagram of differentially expressed genes shared between ABP(D0) and ABP(D6), ABP(D13) or ABP(D21). A rectangular box is used to mark some shared differentially expressed genes, including neuron marker genes (*Tubb3*, *Map2*, *Mapt*) and RGC marker genes (*Pou4f1*, *Gng3*, *Stmn3*, *Nefl*).

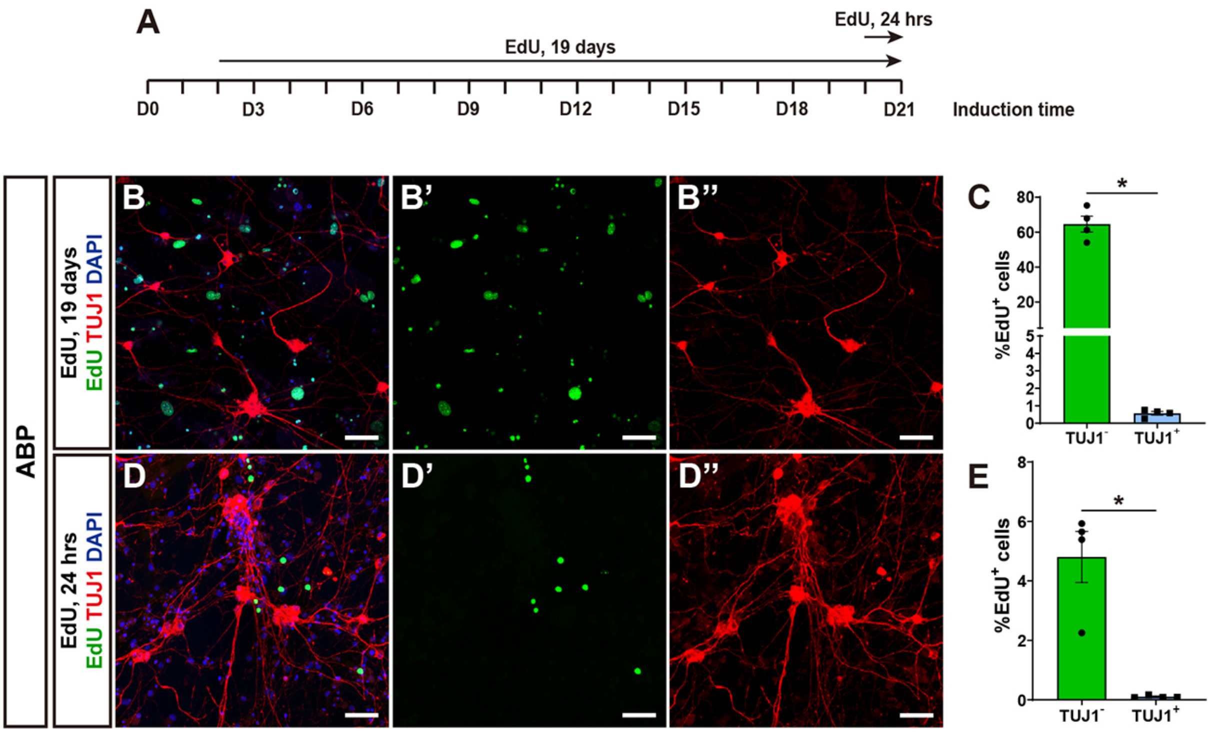

Figure S6. Induction of iRGCs by ABP without undergoing a proliferative intermediate state. (A) Schematic illustration of EdU labeling schedule during the ABP reprogramming process. (B–B'') ABP-transduced MEFs were labeled by EdU for 19 days and co-labeled by fluorescence for both TUJ1 and EdU. Scale bars: 40 µm. (C) Quantification of corresponding EdU-labeled cells in populations of TUJ1-negative and TUJ1-positive cells. Data are presented as mean ± SD (n = 4). The asterisk indicates significance in unpaired two-tailed Student's t-test: *p < 0.0001. (D–D'') ABP-transduced MEFs were labeled by EdU for 24 hours and co-labeled by fluorescence for both TUJ1 and EdU. Scale bars: 40 µm. (E) Quantification of corresponding EdU-labeled cells in populations of TUJ1-negative and TUJ1-positive cells. Data are presented as mean ± SD (n = 4). The asterisk indicates significance in unpaired two-tailed Student's t-test: *p<0.05.

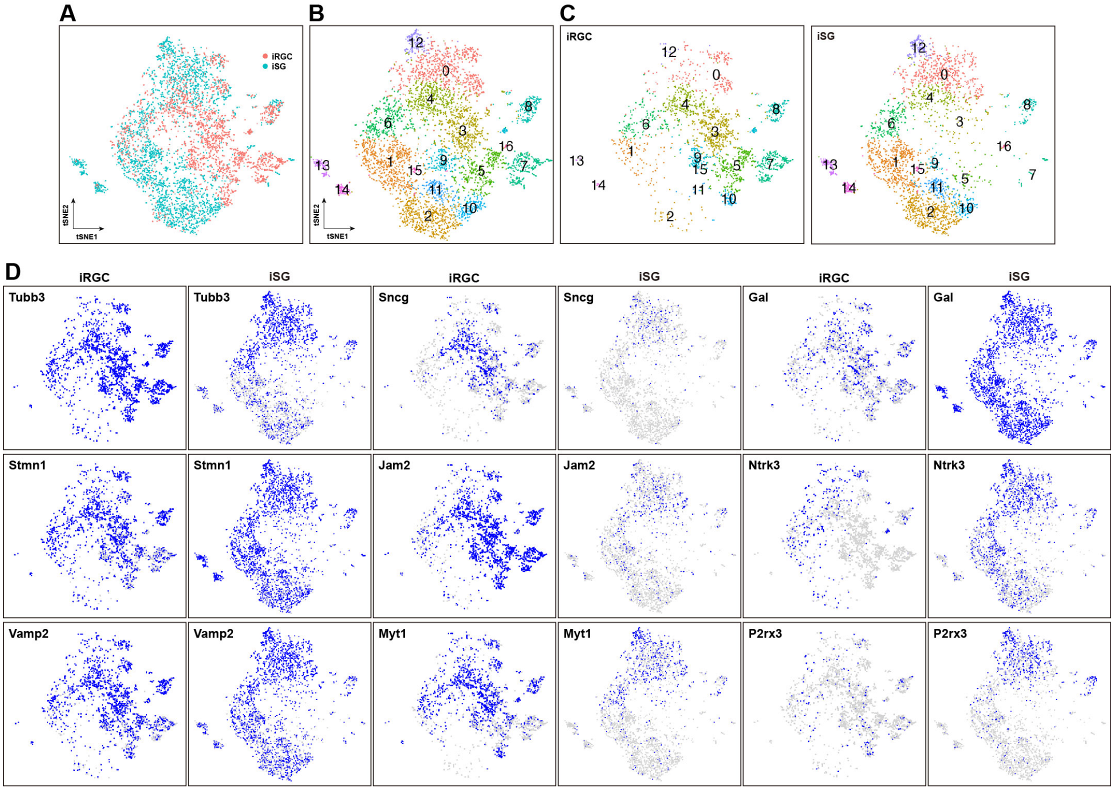

Figure S7. Integrated analysis of scRNA-seq transcriptomes of ABP-induced iRGCs and ABI-induced iSG neurons. (A) t-SNE visualization of the ABP-induced iRGCs and the iSG (induced sensory ganglion) neurons induced by ABI (ASCL1 + BRN3B/3A + ISL1). The accession code of the iSG scRNA-seq dataset is PRJNA597624. (B) t-SNE plot of the 17 cell clusters generated from the integrated iRGC and iSG datasets. (C) Comparison of t-SNE plots of iRGCs and iSG neurons reveals that they form very distinct cell clusters. (D) Split t-SNE plots of iRGCs and iSG neurons colored by expression of the general neuronal marker genes *Tubb3*, *Stmn1* and *Vamp2*, iRGC-specific genes *Sncg*, *Jam2* and *Myt1*, and iSG-specific genes *Gal*, *Ntrk3* and *P2rx3*.

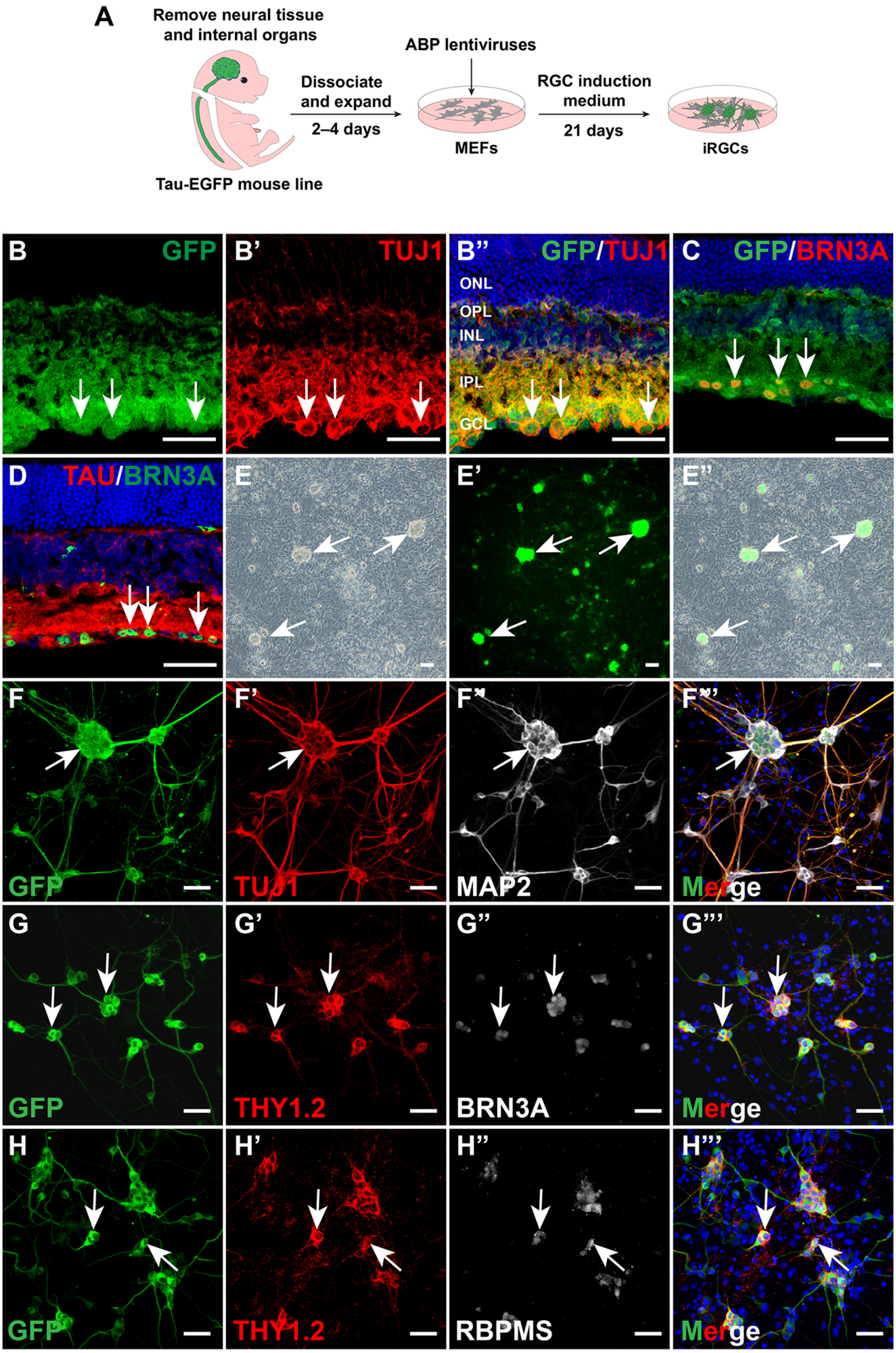
A
Remove neural tissue and internal organs
Tau-EGFP mouse line
Dissociate and expand
2–4 days
ABP lentiviruses
MEFs
RGC induction medium
21 days
iRGCs
B GFP
B' TUJ1
B'' GFP/TUJ1
ONL
OPL
INL
IPL
GCL
C GFP/BRN3A
D TAU/BRN3A
E
E'
E''
F GFP
F' TUJ1
F'' MAP2
F''' Merge
G GFP
G' THY1.2
G'' BRN3A
G''' Merge
H GFP
H' THY1.2
H'' RBPMS
H''' Merge

Figure S8. GFP-tagged iRGCs induced by ABP from Tau-EGFP MEFs. (A) Schematic illustration of the process to induce GFP-tagged iRGCs. (B–B'', C) Adult Tau-EGFP mouse retinal sections were double-immunostained for GFP and TUJ1 or BRN3A. Nuclei were stained with DAPI. Arrows indicate colocalized RGCs. GCL, ganglion cell layer; INL, inner nuclear layer; IPL, inner plexiform layer; ONL, outer nuclear layer; OPL, outer plexiform layer. Scale bars: 40 µm. (D) Adult C57BL/6 mouse retinal sections were double-immunostained for TAU and BRN3A. Nuclei were stained with DAPI. Arrows indicate colocalized RGCs. Scale bars: 40 µm. (E–E'') Corresponding bright-field and fluorescent images of ABP-transduced Tau-EGFP MEFs on D21. Arrows indicate GFP-positive iRGCs. Scale bars: 40 µm. (F–F''', G–G''', H–H''') ABP-induced GFP-tagged iRGCs were triple-immunostained with the indicated antibodies. Nuclei were stained with DAPI. Arrows indicate colocalized cells. Scale bars, 40 µm.

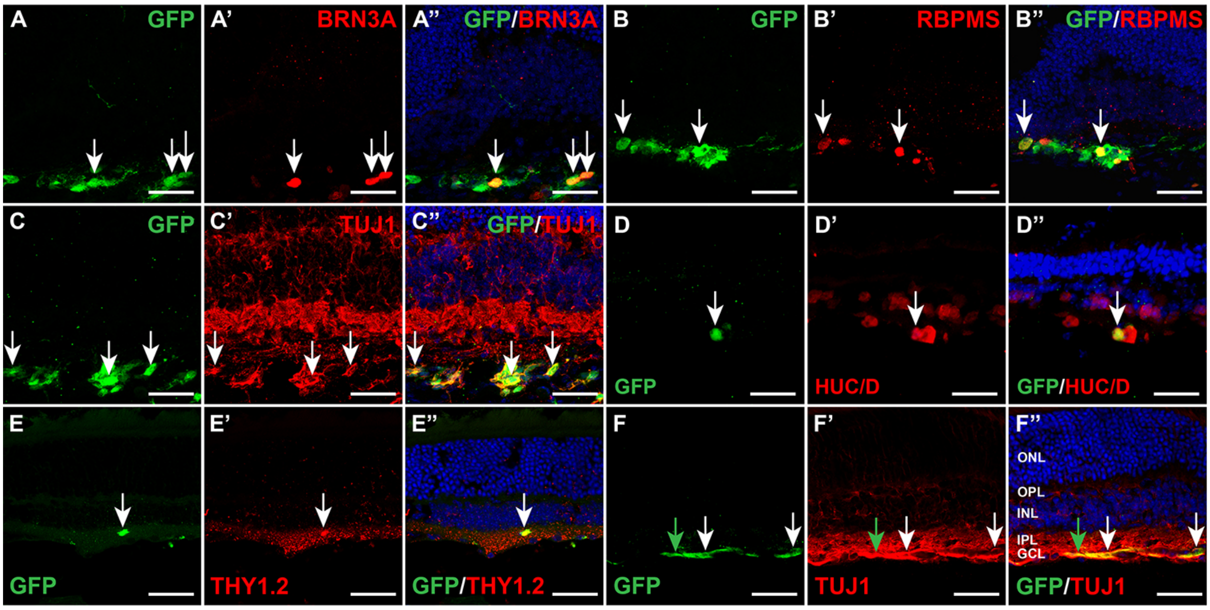

Figure S9. Immunofluorescence labeling showing the survival and integration of ABP-indued GFP-tagged iRGCs in retinal explants and NMDA-damaged retinas. (A–A", B–B", C–C'') Sections of retinal explants transplanted with GFP-tagged iRGCs were double-immunostained with the indicated antibodies. Nuclei were stained with DAPI. White arrows indicate colocalized iRGCs. Scale bars, 40 µm. (D–D", E–E", F–F'') Sections of NMDA-damaged retinas transplanted with GFP-tagged iRGCs were double-immunostained with the indicated antibodies. Nuclei were stained with DAPI. White arrows indicate colocalized iRGCs and green ones indicate colocalized iRGC fibers. GCL, ganglion cell layer; INL, inner nuclear layer; IPL, inner plexiform layer; ONL, outer nuclear layer; OPL, outer plexiform layer;. Scale bars, 40 µm.

Table S1. Primary Antibodies

Antibodies	Source (Cat#)	Host	Dilution
Tubulin β III (TUJ1)	Millipore (MAB5564)	Mouse	1:2000
beta III Tubulin (TUJ1)	abcam (ab18207)	Rabbit	1:2000
Brn-3	Santa Cruz Biotechnology (sc-390780)	Mouse	1:200
Brn-3a	Millipore (MAB1585)	Mouse	1:500
Brn-3a	Millipore (AB5945)	Rabbit	1:1000
RBPMS	Novus Biologicals (NBP2-20112)	Rabbit	1:500
CD90.2 (Thy1.2)	BD Pharmingen™ (550543)	Rat	1:200
gamma Synuclein/SNCG	abcam (ab55424)	Rabbit	1:100
Islet 1 (ISL1)	abcam (ab20670)	Rabbit	1:1000
HuC/HuD	Invitrogen (A-21271)	Mouse	1:500
Vimentin	abcam (ab92547)	Rabbit	1:500
MAP2	Millipore (AB5543)	Chicken	1:2000
MAP2 (2a+2b)	Sigma (M1406)	Mouse	1:2000
MAP2	abcam (ab32454)	Rabbit	1:2000
GFP	abcam (ab6673)	Goat	1:2000
Synapsin1 (SYN1)	Synaptic Systems (106011)	Mouse	1:2000
Tau	Synaptic Systems (314003)	Rabbit	1:2000
VGLUT1	Synaptic Systems (135303)	Rabbit	1:500
VGLUT2	Synaptic Systems (135403)	Rabbit	1:500
VGLUT3	Sigma (SAB5200312)	Mouse	1:500
Tyrosine Hydroxylase (TH)	Millipore (MAB318)	Mouse	1:500
GABA	Sigma (A2052)	Rabbit	1:1000

Table S2. qRT-PCR Primers

Gene	5’ Primer	3’ Primer
Gapdh	CGTGCCGCCTGGAGAAACCTG	GAGTGGGAGTTGCTGTTGAAGTCGC
Tuj1 (Tubb3)	AACGCATCTCGGAGCAGTTCACA	CCGATTCCTCGTCATCATCTTCATACATC
Brn3a (Pou4f1)	CGCGCAGCGTGAGAAAATG	CGGGGTTGTACGGCAAAATAG
Sncg	CCAAGCAGGGAGTAACGGAG	GGTTCCAAGTCCTCCTTGCG
Thy1.2 (Thy1)	GGGAGTCCAGAATCCAAGTCG	TCCAGGCGAAGGTTTTGGTT
Isl1	TACGTGCTTTGTTAGGGATGGG	CTGCACTTGGCGCATTTGAT
en-Isl1	CGGAAGAAACCAGCCTCAGT	GGGCATGCTTAAGAGACCCA
Rbpms	TGACAGTCGCTCAGAAGCAGAG	GCCATCTTCGTGTTTGCCTTAGC
HuC	GAGTCAAGAGTCCCCTGTCG	TCATGGTGACGAAGCCGAAA
Map2	TCCCCAGCTACTCCTAAGCA	AGAGCCACATTTGGATGTCA
Syn1	GCCCAGATGGTTCGACTACAC	TGTTGGTTGTCTACCTTGACCT
Pou4f3	CGACGCCACCTACCATACC	CCCTGATGTACCGCGTGAT
Gap43	AGCCAAGGAGGAGCCTAAAC	TTCGTCTACAGCGTCTTTCTCC
Nefm	ACAGCTCGGCTATGCTCAG	CGGGACAGTTTGTAGTCGCC
**Vimentin (*Vim*)**	CTTGAACGGAAAGTGGAATCCT	GTCAGGCTTGGAAACGTCC
Vglut1	GAGGAGCGCAAATACATTGAGG	CCAGGGTGTGTTAAACTTCGTAA
Vglut2	GCGGAGGCAAAGTTATCAAG	CCTGGAATCTGGGTGATGAT
Vglut3	CGGTATGTTTGGGATTATTTGGT	CATAGACAGGCAAGGATGTGAAA
Th	GTGCCAGAGAGGACAAGGTTC	CGATACGCCTGGTCAGAGA
Glyt1	TACCTCTGCTATCGCAACGG	CAAACTGGCCGAAGGAAAGC
Gad1	GGCCTGAAGATCTGTGGCTT	CCCGTTCTTAGCTGGAAGCA

Other Supplementary Materials for this manuscript:

Table S3 (Microsoft Excel format). List of differentially expressed genes (fold change ≥ 2 and p-value < 0.05) between ABP-transduced and GFP-transduced MEFs (D21) as determined by bulk RNA-seq analysis.

Videos S1 and S2. MEFs were induced by ABP (ASCL1, BRN3B and PAX6) or A (ASCL1) for 10 days, and then recorded by long-term time-lapse microscopy for 50 hours. The recording results showed that ABP-induced neurons would self-organize into neuronal clusters (Video S1), while no self-organization was observed for ASCL1-induced neurons (Video S2).
